# Towards Improving Embryo Prioritization: Parallel Next Generation Sequencing of DNA and RNA from a Single Trophectoderm Biopsy

**DOI:** 10.1038/s41598-019-39111-7

**Published:** 2019-02-27

**Authors:** Noga Fuchs Weizman, Brandon A. Wyse, Ran Antes, Zenon Ibarrientos, Mugundhine Sangaralingam, Gelareh Motamedi, Valeriy Kuznyetsov, Svetlana Madjunkova, Clifford L. Librach

**Affiliations:** 1 CReATe Fertility Centre, Toronto, Canada; 2 Department of Obstetrics and Gynecology, University of Toronto, Toronto, ON Canada; 3 Department of Physiology, University of Toronto, Toronto, ON Canada; 4 Department of Gynecology, Women’s College Hospital, Toronto, ON Canada

## Abstract

Improved embryo prioritization is crucial in optimizing the results in assisted reproduction, especially in light of increasing utilization of elective single embryo transfers. Embryo prioritization is currently based on morphological criteria and in some cases incorporates preimplantation genetic testing for aneuploidy (PGT-A). Recent technological advances have enabled parallel genomic and transcriptomic assessment of a single cell. Adding transcriptomic analysis to PGT-A holds promise for better understanding early embryonic development and implantation, and for enhancing available embryo prioritization tools. Our aim was to develop a platform for parallel genomic and transcriptomic sequencing of a single trophectoderm (TE) biopsy, that could later be correlated with clinical outcomes. Twenty-five embryos donated for research were utilized; eight for initial development and optimization of our method, and seventeen to demonstrate clinical safety and reproducibility of this method. Our method achieved 100% concordance for ploidy status with that achieved by the classic PGT-A. All sequencing data exceeded quality control metrics. Transcriptomic sequencing data was sufficient for performing differential expression (DE) analysis. All biopsies expressed specific TE markers, further validating the accuracy of our method. Using PCA, samples clustered in euploid and aneuploid aggregates, highlighting the importance of controlling for ploidy in every transcriptomic assessment.

## Introduction

With increasing practice of elective single embryo transfer (eSET) in assisted reproductive technologies (ART), as means to avoid multiple gestations and their associated complications, improved tools for embryo prioritization are crucial for maximizing pregnancy rates per embryo transfer. Previous partially successful efforts for embryo prioritization include, but are not limited to, morphological and/or morphokinetic criteria^1–5^ as well as the ploidy status of an embryo^6–8^. However, both are of limited value and there is still a need for better embryo prioritization tools that will improve the efficiency of eSET.

Implantation failure and/or early development failure are believed to be due to a range of factors including chromosomal abnormalities, asynchrony between embryo development and uterine receptivity, and factors associated with treatment interventions and techniques^9^. Several molecular processes and pathways involved in the early stages of development and implantation have been characterized including cell cycle regulation, DNA repair, apoptosis, maintenance of accurate chromosomal segregation, and assembly of the cytoskeleton^10–12^. However, the complete molecular dialogue between the maternal and the fetal components leading to implantation, and in particular the role of the blastocyst still remains poorly understood^13^. Defining what differs between viable and non-viable embryos and specifically, understanding the process of implantation and early development is important for optimizing fertility treatment outcomes for the entire infertile population^14^.

Due to the inherent challenges of studying human embryos, most available knowledge of the molecular basis of preimplantation embryonic development comes from gene expression studies on mouse, bovine, and non-human primate embryos^15–19^. Studies performed to date on human embryos suffer from several drawbacks. Jones *et al*. have performed a full transcriptomic analysis of embryos in early developmental stages^9^; however, due to technological barriers at the time, they had to pool several embryos to yield sufficient mRNA for sequencing, which impedes our ability to draw conclusions on a single embryo level^9^. In 2013, Yan *et al*. were the first to perform transcriptome analysis by Next Generation Sequencing (NGS) on single cells derived from human embryos^20^, followed by Petropolous *et al*. that aimed to better define lineage segregation at the first stages of development using single cells from dissociated embryos^21^. While it has been established in many species that ploidy alters the transcriptome^22–25^ both of the above were not able to control for the ploidy status of their studied embryos, which limits the validity of their findings. In a hallmark study by Kirkegaard *et al*., the gene expression was further correlated with clinical outcomes; however, their findings were also limited by the lack of control for the ploidy status of the tested embryos^14^. Finally, Marine *et al*. have recently published a study examining cellular pathways that are activated during early embryonic stages, while controlling for the ploidy status, however they have done so via RT-qPCR, which is highly selective and, by focusing on a given set of genes, can introduce significant bias to the data^12^.

Enhancing our understanding of the process of early embryonic development and implantation can, in turn, aid in designing the ultimate embryo prioritization tool. In order to do so, one must correlate the transcriptome with clinical outcomes while controlling for embryo ploidy. Such a dataset would help delineate viable euploid embryos from euploid embryos that are not. In order to correlate these research findings with clinical outcomes, such studies must be integrated into the current clinical workflow for the analysis of embryo ploidy, without harming it or making it more cumbersome.

In the last 10 years, there has been a significant push to develop single cell RNAseq methods^26–32^. Single cell-omics presents many challenges, including limited starting material while at the same time generating a large amount of data which exponentially increases the resources required to analyze, consolidate, and interpret these large datasets. Subsequently, advances in bioinformatic tools which can better control for increased sequencing noise, batch effects, and entanglement of biological and technical variability, have made it more feasible to integrate multiple layers of data (RNAseq, DNA methylation, histone modifications etc.)^32–37^. These advancements, in turn, have driven the development of several methods for parallel sequencing of both mRNA and gDNA from the same cell or cell population. Some methods introduce additional reverse transcription and amplification steps^24^, some require magnetic bead separation steps prior to the gDNA isolation and amplification^25^, and others rely on physical separation of the nucleus from the cytoplasm^38^. The most recently described simultaneous mRNA and gDNA sequencing method, termed SIDR-Seq, introduces a step of antibody-conjugated magnetic microbeads separation prior to amplification^39^. All the aforementioned methods were not applicable for the purpose of this study since they introduce additional steps into the current clinical workflow of embryo ploidy status determination, which is commonly referred to as preimplantation genetic testing for aneuploidy (PGT-A). Using the aforementioned methods would require extensive validation and training while making the process more cumbersome and costly.

Here we introduce PGT-AT (Preimplantation Genetic Testing for Aneuploidy and Transcriptome), in which we create a clinically applicable tool for simultaneous assessment of chromosomal copy number by low pass whole genome sequencing, and transcriptomic profile using whole transcriptome RNAseq. For this proof of concept study, embryos donated for research with known ploidy were selected (either aneuploid or euploid embryos harboring a disease causing gene mutation, deeming them not desirable for transfer). Once both the safety and the feasibility of this method are established, the transcriptome of euploid embryos destined for transfer will be assessed and the gene expression correlated with clinical outcomes.

## Results

### Blastocyst collection and biopsy

Twenty-five blastocysts were biopsied for this study from 21 patients (see Table 1 for patient demographics). Eight were used for lysis and sequencing library optimization, and 17 were used for simultaneous sequencing of gDNA and mRNA. Tables 2 and 3 present details on the analyzed embryos, biopsies taken, and methods used.

**Table 1:** Characteristics of patients included in the study.

Patient Characteristics	Mean	SEM	Range
Age (years)	35	1.7	21 to 43
BMI (kg/m^2^)	23.55	1.30	20 to 32
AMH (pmol/L)	29.75	5.56	3 to 70
Number of MII eggs	10	1.5	5 to 22
Sperm Total Motility Count (millions)	111	30.1	9 to 340
Sperm DNA Fragmentation Index (%)	21.71	5.95	8 to 47
Fertilization Rate (%)	73	6	33 to 90
Number of day 3 embryos	8	1.7	3 to 18
Number of day 5 embryos	3	0.6	0 to 7

SEM – Standard Error of Mean.

BMI – Body Mass Index.

AMH – Anti-Mullerian Hormone.

MII - mature metaphase II oocytes.

**Table 2:** Optimization of lysis method – VeriSeq® next generation sequencing results from chromosomal aberration analysis and sequencing quality metrics.

Blastocyst number	TE-A - Unsplit, Control Sample				TE-B - Split, PGT-AT Sample			
	Ploidy	WGA-DNA Concentration (ng/ul)	Number of Reads	Overall noise (DLR)	Ploidy	WGA-DNA Concentration (ng/ul)	Number of Reads	Overall noise (DLR)
B1^a^	XX: (−5q14.1-qTerm, 100 Mb, 20%)	36.8	599296	0.16	Noisy Signal	25.1	1092118	0.43
B2^a^	XY: (+1, −3, +21)	39.1	759530	0.22	Noisy Signal	26.9	490957	4.38
B3^a^	XX: (+13)	30.3	544132	0.22	Noisy Signal	31.5	215583	485.48
B4^a^	XY: Euploid	36.0	480716	0.20	Noisy Signal	21.3	188363	1.21
B5^b^	XX: (+19)	32.1	328148	0.23	XX: (+19)	30.4	638468	0.16
B6^b^	XY: Euploid	29.5	565795	0.17	XY: Euploid	28.4	365915	0.21
B7^b^	XY: Euploid	38.4	555431	0.16	XY: Euploid	31.9	473225	0.21
B8^b^	XX: Euploid	27.0	928647	0.14	XX: Euploid	32.2	613360	0.18

DLR–Derivative Log ratio; ^a^Samples lysed using the SMART-seq® lysis method; ^b^Samples lysed using the SurePlex® lysis method.

**Table 3:** VeriSeq® next generation sequencing results from chromosomal aberration analysis and sequencing quality metrics from simultaneous mRNA/gDNA sequencing using the novel PGT-AT method.

Blastocyst number	TE-A - Clinical Sample				TE-B - PGT-AT Sample			
	Ploidy	WGA-DNA Concentration (ng/ul)	Number of Reads	Overall noise (DLR)	Ploidy	WGA-DNA Concentration (ng/ul)	Number of Reads	Overall noise (DLR)
B9	XX: −16	38.7	440175	0.18	XX: −16	45.6	643561	0.18
B10^a^	XX: Euploid	36.5	1599844	0.29	XX: Euploid	48.1	639856	0.20
B11 ^a^	XY: Euploid	41.6	979390	0.20	XY: Euploid	40.5	628025	0.28
B12 ^a^	XX: Euploid	37.6	1006623	0.23	XX: Euploid	48.3	456403	0.21
B13 ^a^	XX: Euploid	35.2	123950	0.32	XX: Euploid	46.1	414963	0.20
B14 ^a^	XX: Euploid	43.7	191719	0.26	XX: Euploid	46.3	537101	0.17
B15 ^a^	XY: Euploid	40.4	1272213	0.19	XY: Euploid	45.5	417477	0.20
B16 ^a^	XX: Euploid	38.1	1039379	0.18	XX: Euploid	42.3	635682	0.17
B17	XX:+16	45.6	550328	0.17	XX:+16	49.3	462388	0.21
B18	XX: −16	47.2	461997	0.17	XX: −16	50.1	493227	0.20
B19	XY: −16	42.3	599572	0.17	XY: −16	47.5	465943	0.19
B20	XX: −16	35.7	699078	0.15	XX: −16	50.1	500694	0.22
B21	XX: −16	36.2	804292	0.19	XX: −16	49.6	457069	0.25
B22	XY:+16	46.8	464953	0.20	XY:+16	49.1	465756	0.21
B23	X0	47.8	770087	0.25	X0	47.9	570588	0.17
B24	XX:+16	39.1	460520	0.19	XX:+16	48.2	655510	0.17
B25	X0	40.9	567071	0.15	X0	77.6	282556	0.26

DLR–Derivative Log ratio, TE-trophectoderm ^a^TE-A Clinical sample amplified with RepliG® (QIAGEN, Germany). TE-B PGT-AT sample processed using the optimized PGT-AT method.

### Optimized TE biopsy cell lysis

**Figure 1:**
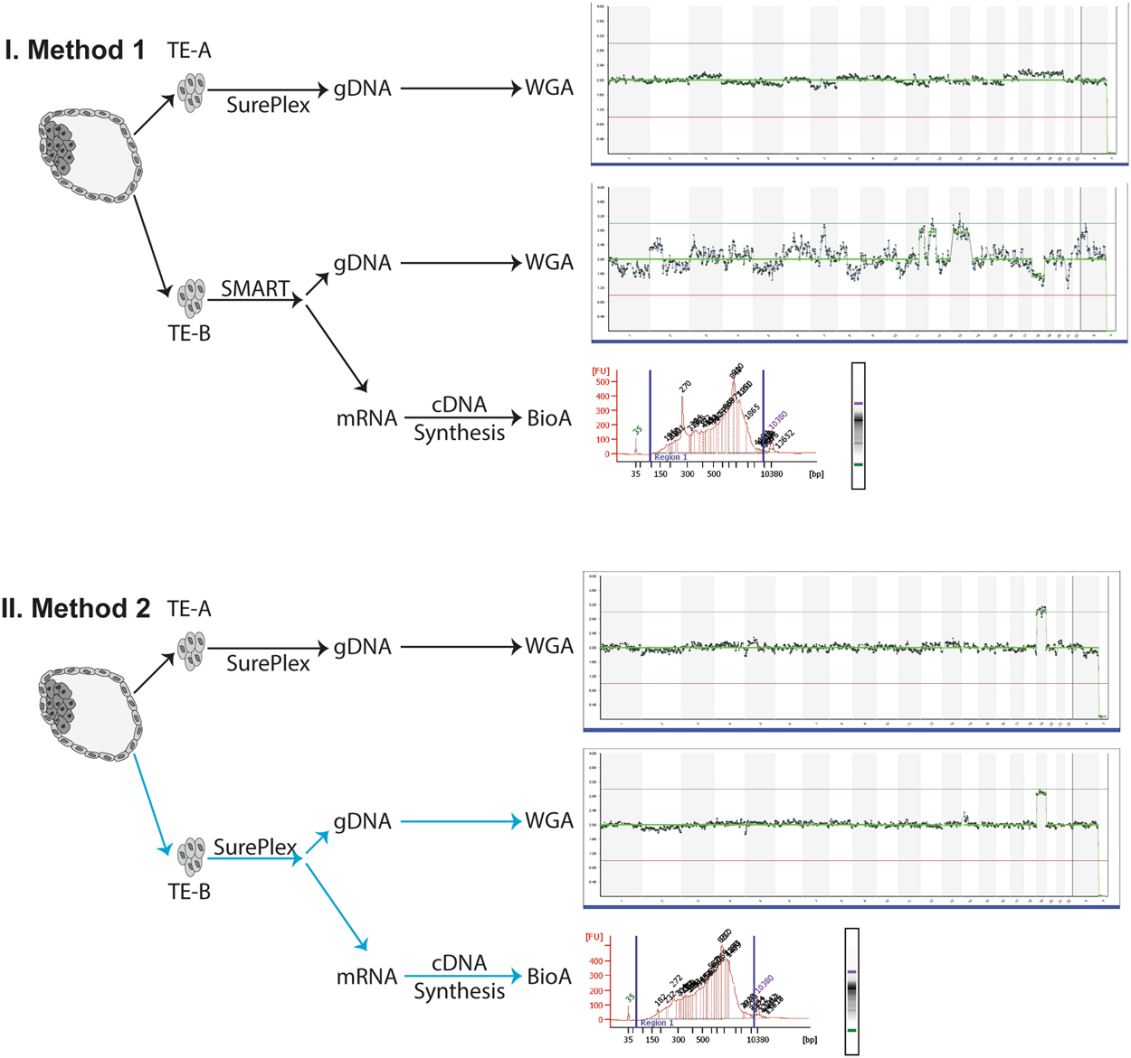
Detailed workflow of the cell lysis optimization to obtain both high-quality gDNA and mRNA from the same TE biopsy. TE-A is the “clinically representative” control biopsy, lysed and processed using the standard clinical workflow for PGT-A. TE-B is the test biopsy, where cells are lysed with either SMART (Method 1) or SurePlex® (Method 2) kits. The lysate was split and processed according to the standard SurePlex® protocol for gDNA amplification, or the standard SMART-seq® protocol for cDNA synthesis. Lysis of biopsied cells with SurePlex® yields high-quality gDNA and mRNA. From each blastocyst cDNA was synthesized, amplified, and its integrity/quality was assessed by BioAnalyzer 2100 (Agilent Technologies, CA). All samples, regardless of lysis method, produced high-quality cDNA. However, only the sample lysed using SurePlex® (Method 2) produced both high-quality cDNA and gDNA which passed all clinical quality control metrics after NGS using VeriSeq® kit (highlighted with blue arrows).

As described in Fig. 1 we compared the performance of two different cell lysis methods to obtain gDNA and mRNA from the same sample (n = 8): I. SMART-seq® (Takara BioInc, CA) or II. SurePlex® kit (Illumina, CA). Biopsied cells lysed using the SMART-seq® protocol (I) (Takara BioInc, CA) yielded cDNA of high-quality and quantity, measured by BioAnalyzer 2100 (Agilent Technologies, CA) and fluorometer, respectively. The quantity of gDNA from this lysis approach was in the expected range (Table 2), however, the integrity was affected which resulted in noisy low pass whole genome next generation sequencing (NGS) results using VeriSeq® kit (496,755 average reads, range of noise [derivative log ratio–DLR] 0.43–485.48) (Fig. 1). The SurePlex® lysis method (II) yielded high-quality and quantity of cDNA and gDNA yielding high-quality NGS results using VeriSeq® kit (522,668 average reads, range of noise [DLR] 0.16–0.21). Based on these findings, we established our PGT-AT method: (1) Single TE biopsy, (2) SurePlex® cell lysis mix, (3) Lysate splitting (4) Simultaneous, independent gDNA amplification and cDNA synthesis (indicated in Fig. 1 with blue arrows). Moving forward, we applied our method to test 17 additional embryos.

### Concordance of embryo ploidy status between standard clinical PGT-A and the novel PGT-AT methods

Table 3 summarizes the embryo ploidy status of standard clinical PGT-A and the corresponding ploidy status obtained from additional TE biopsies using the novel PGT-AT method from 17 embryos donated for research. There was 100% concordance of copy number variation (CNV) analysis by gDNA low pass whole genome sequencing, between standard clinical PGT-A results and PGT-AT results from the same blastocysts (Table 3).

### The TE transcriptome obtained with PGT-AT was of high quality

**Figure 2:**
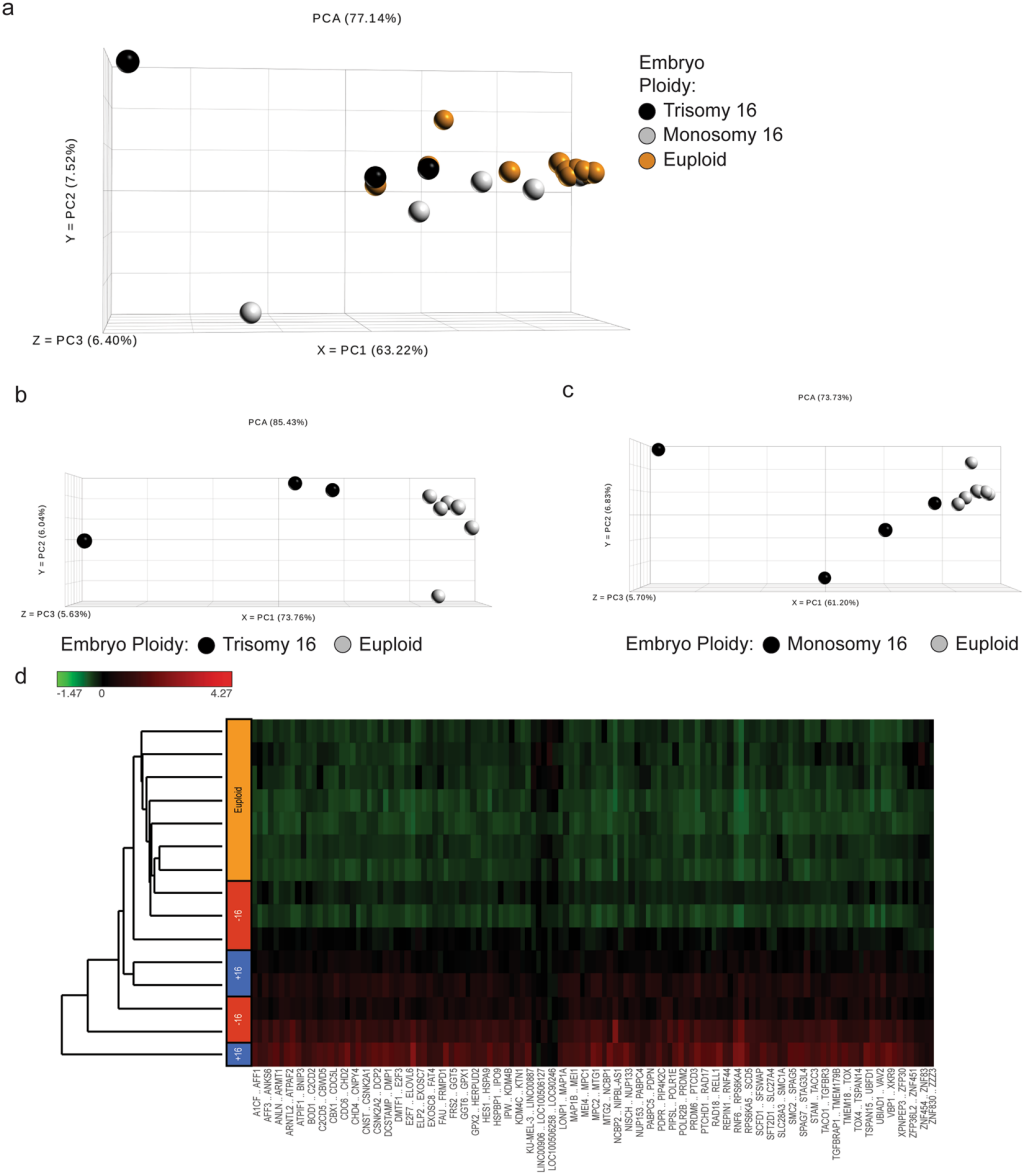
Principal component analysis and hierarchical clustering of trophectoderm transcriptome. (**a**) Principal component analysis of all blastocysts (trisomy/monosomy 16, n = 8 and euploid, n = 7) shows significant separation along PC1 and PC2 by ploidy. This is further exaggerated when comparing trisomy (n = 3) or monosomy 16 (n = 5) alone to euploid (n = 7), (**b**–**d**) Samples also cluster under unsupervised hierarchical clustering when plotting standardized expression values. Here, samples are on the rows and features on the columns (red indicating high standardized expression, green indicating low standardized expression).

All tested samples produced sufficient quantity and high-quality cDNA for RNAseq, as per BioAnalyzer 2100 (Agilent Technologies, CA) (see Supplementary Fig. S2), enabling us to conduct gene expression profiling. Principal component analysis (PCA) revealed tight clustering of the euploid samples harboring single-gene mutations, along principal component one (PC1), accounting for 63.2% of the variability between all the depicted samples. The aneuploid samples in our study were scattered along the PC2 axis, which accounted for 7.5% of the variability between the depicted samples (Fig. 2). Hierarchical clustering based on normalized read counts further demonstrated the significant differences between euploid embryos harboring single gene mutations in our study, and embryos with either monosomy 16 (M16) or trisomy 16 (T16) (Fig. 2d).

### RNA transcripts differ in accordance with the ploidy status of the embryo

**Figure 3:**
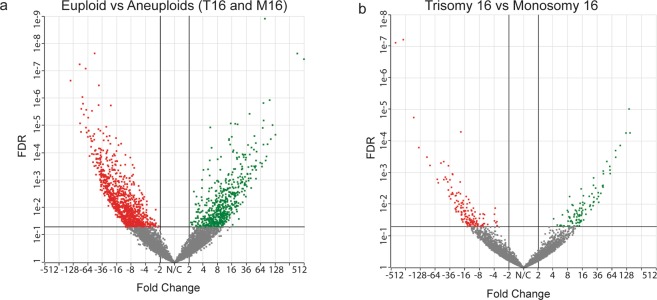
Differential expression analysis between TE biopsies from euploid and aneuploid blastocysts. (**a**) When comparing euploid vs aneuploid (T16 and M16 together) biopsies using DEseq. 2, 1574 transcripts were differentially expressed (1001 down-regulated and 573 up-regulated). (**b**) When comparing trisomy 16 vs monosomy 16 using DEseq. 2, 251 transcripts were differentially expressed (155 down-regulated and 96 up-regulated). Red indicates significantly downregulated (FC < −2) and green significantly upregulated genes (FC > 2) at FDR < 0.05.

Several genes that are active in placentation were differentially expressed between euploid embryos harboring single gene mutations and aneuploid embryos including *FADS1*(−8.9 fold), *PGK1*(−7.7 fold), and *PPAT* (−11.7 fold). When comparing euploid embryos to either monosomy or trisomy 16 embryos; genes associated with mitochondrial respiration (i.e. *STOML2*), angiogenesis (i.e. *EMP2*), and cell migration and invasion (i.e. *PTTG1*) were differentially expressed. Several genes involved in cell cycle regulation, DNA damage repair (*RHOBTB1* and *DDX19B, GDF15*), and cell signaling (*FGFR2*) were differentially expressed between embryos with T16 and M16. All differentially expressed genes across different comparisons are presented in Fig. 3 and Supplementary Tables S2–5. qPCR validation of five TE specific gene markers revealed similar expression by both NGS and qPCR (see Supplementary Fig. S3).

### Euploid embryos significantly down-regulate pathways involved in energy metabolism, transcription, and translation

**Figure 4:**
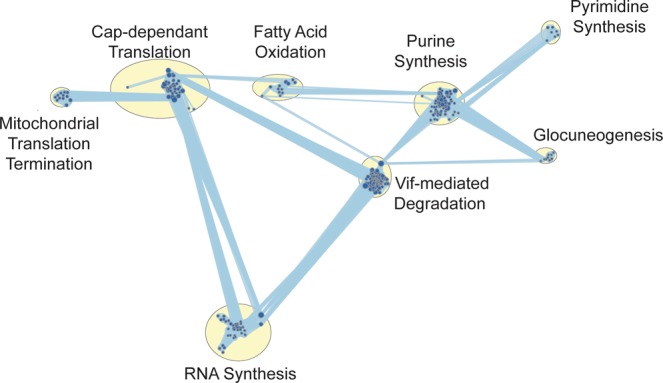
Pathway analysis of significantly differentially expressed genes. GSEA (Gene Set Enrichment Analysis) reveals that euploid embryos significantly downregulate pathways involved in energy metabolism, transcription, and translation. When comparing euploid to all aneuploid samples using GSEA, 156 gene sets were significantly enriched for downregulated genes at FDR < 0.1. The size of the node corresponds to the number of genes in each gene set.

When comparing TE samples from euploid embryos with TE samples from aneuploid embryos, 156 gene sets were enriched for down-regulated genes in the euploid cohort (FDR < 0.1). Most notably, pathways involved in energy metabolism (fatty acid oxidation, gluconeogenesis, and mitochondrial translation termination) were significantly enriched in down-regulated genes in the euploid cohort (Fig. 4). Moreover, pathways involved in nucleotide synthesis, RNA transcription and protein translation were also enriched in down-regulated genes in the euploid cohort (Fig. 4).

## Discussion

In this work we have shown that it is clinically feasible to explore the transcriptome of an embryo alongside its ploidy status from a single trophectoderm biopsy of 4–6 cells. Notably, the addition of RNAseq did not interfere with the workflow of preimplantation genetic testing for aneuploidy, as it is performed in clinics, nor did it significantly alter the clinical quality control metrics (Table 3). A concordance of 100% makes us confident that this method could potentially be introduced into clinical use alongside the current workflow of standard clinical PGT-A. The high level of concordance is a testament to the safety of this method and the consistency across all 17 samples in this study is proof of its reproducibility.

By establishing this method, we have created a novel platform allowing genomic and transcriptomic embryo assessment that could be seamlessly integrated into the current PGT-A workflow. Pending future correlation with outcomes, this novel platform has the potential to not only improve our embryo prioritization abilities, but also to provide a valuable research tool that will enhance our understanding of the first stages of embryonic development and implantation. By gaining insight into the transcriptomics of developing embryos destined for transfer, both embryo prioritization and treatment choices could be optimized.

To date, all the available methods for parallel mRNA and gDNA sequencing on a single cell level were not applicable for the purpose of our study since they introduce additional steps into the current workflow of PGT-A and would require extensive validation and training prior to implementation. Furthermore, they are bound to make the process more cumbersome and costly.

We developed an approach that allows for the simultaneous sequencing of mRNA and gDNA from a low number of cells, here termed PGT-AT, which can be immediately incorporated into the current clinical workflow used for ploidy testing. This will enable us to move forward using PGT-AT without the need for extensive clinical validation of the ploidy assessment step. Furthermore, to facilitate the widespread adoption of PGT-AT as a research tool, we chose a transcriptomic analysis approach that is easy to implement, sensitive, time-efficient, reproducible, and inexpensive. The method that best fit the above criteria for the low-input RNAseq arm of our project was a commercial development of SMART-seq^27^ (Takara Biosciences Inc.). By modifying the cell lysis step, we maintained the integrity of mRNA while additionally sequencing gDNA at a clinically acceptable quality (Fig. 1).

Previous studies correlating embryos’ transcriptome to assisted reproductive technologies (ART) outcomes in humans have done so without controlling for ploidy^9,14,21^. Our current method not only employs next generation sequencing but is also the first study to control for ploidy of the embryo when analyzing the full transcriptome. The importance of this ability is partially demonstrated by a 122-fold increase in the expression of *DDX19B* (involved in DNA repair) in T16 versus M16. This gene is located on the q-arm of chromosome 16 and the increased expression in T16 might simply be a result of the increased gene copy number due to trisomy and not necessarily biologically significant. Furthermore, it is well established that aneuploidy is often associated with chromosome and sequence rearrangements, the activation of transposons, the amplification or elimination of highly repetitive sequences, and changes in the regulation of gene expression, thus when comparing samples of different ploidy the correlations are not linear^22^. When comparing between the transcriptomics of euploid and aneuploid embryos, our findings echo what is known in the literature; ploidy dramatically affects the transcriptome^22–25^. This can be clearly deduced by principal component analysis and hierarchical clustering analysis (Fig. 2). Therefore, any study attempting at delineating embryonic development via RNAseq should control for the ploidy status of the embryo so that the findings are valid. We used PCA as a tool for initial exploratory data analysis; with the plan of conducting further t-distributed stochastic neighbor embedding (tSNE) analysis at later stages when exploring specific genes that seem to correlate with outcomes on larger datasets.

When comparing pathways between euploid embryos and aneuploid embryos, the euploid embryos were enriched in several down-regulated pathways involved in energy metabolism (fatty acid oxidation, gluconeogenesis, and mitochondrial translation termination) nucleotide synthesis, RNA transcription and protein translation (Fig. 4). This is in agreement with the quiet embryo hypothesis, which states that embryo survival is increased by minimizing metabolism during the first stages of development^40^. These findings are further strengthened by previous studies that compared metabolite usage in different commercial embryo culture media^40–42^. *STOML2*, which is a key regulator of mitochondrial respiration, was up regulated in trisomy 16 embryos when compared with euploid embryos, further supporting the quiet embryo hypothesis. Interestingly, *FADS1*, involved in lipid metabolism and turnover, was up regulated in euploid embryos when compared with aneuploid embryos in our cohort. *FADS1* has been shown to be abundant in elongating conceptus in sheep, and to correlate with reproductive potential in cattle^43,44^. Three genes important for implantation: *PGK1, PPAT*, and *EMP2*, which modulate angiogenesis and cell invasion, were differentially expressed between euploid and aneuploid embryos in our cohort. Notably, previous studies on *EMP2* suggest that it may regulate implantation by orchestrating the surface expression of integrins and other membrane proteins^45^. *EMP2* was previously shown to be significantly reduced in both villous and extra-villous trophoblast populations in placentas of pregnancies with fetal intrauterine growth restriction^46^. When comparing our data to available data in the literature it should be noted that many factors alter the transcripts that are detected by sequencing and the level of their expression, thus making it challenging to compare different datasets. Some of the factors that contribute to variability between different datasets are the method for obtaining the sample (either dissociating the embryos and picking up individual cells or utilizing TE biopsies)^47^, method of fertilization (intra-cytoplasmic sperm injection or *in vitro* fertilization), culture conditions (open system vs. closed culturing systems), fresh versus vitrified-thawed embryos, and parental demographics^14,20^. It is important to highlight that this study was not designed to delineate the differences between viable and non-viable embryos. However, datasets such as this one provides the groundwork/benchmark of the expected transcriptomic profile of TE cells at a specific time point.

The fields’ knowledge of the human embryo transcriptome has been restricted by limited access to human embryo research material and further complicated by the small amounts of available genetic material within one embryo. Most studies to date have predominantly relied on older techniques, which have several limitations such as limited detection ability, high background noise levels, and inherent bias. Furthermore, the most up to date studies have failed to control for ploidy status and have not incorporated their technique with what is available today in the clinical setting. This study has managed to improve on all these aspects by developing a method that can be seamlessly incorporated into the clinical workflow without impacting the accuracy of aneuploidy detection by PGT-A. This allows one to reliably gain new insight into the transcriptome of embryos while controlling for ploidy and gives one the ability to correlate an embryo’s transcriptome with clinical outcome, such as implantation rate and live birth rate.

Recent work in bovine and murine models has revealed marked transcriptomic heterogeneity among single cells isolated from the trophectoderm^48,49^. Furthermore, this heterogeneity is particularly enhanced during embryonic genome activation and early gastrulation^49,50^. Chromosome instability is common in human cleavage-stage embryos^50,51^. Several studies in human embryos have demonstrated transcriptomic heterogeneity^21,52,53^, that appears to occur alongside enhanced transcriptional noise prior to cell fate decisions, possibly aiding polarization^54–57^.

At this point we can be confident that our approach will reduce the heterogeneity seen with single-cell genomic or transcriptome analysis under assumption that our sample represents the relative profiles of 4–6 trophectoderm cells. What remains to be evaluated is the impact of potential “residual” heterogeneity on our ability to use the transcriptome of a 4–6 cell TE biopsy as a prediction tool for embryo development and implantation. It is also possible that the level of this heterogeneity may in fact prove to be a good metric for assessing the development potential of a blastocyst and may give an indication of future successful gastrulation^20,49,57^. We plan to address these issues and incorporate expression variability in the analysis approach in a future clinical study aimed at correlating transcriptomic characteristics with clinical outcomes^58–60^.

Applying PGT-AT in prospective studies will allow for interrogation of different clinical scenarios and treatments with the transcriptome of the developing blast, and its potential to develop into a healthy baby. Our future research will focus on exploring in detail the validity of the embryo transcriptome as a biomarker for embryo implantation and development, with the ultimate goal of improving embryo prioritization as well as overall outcomes in ART. Differentially expressed genes between blastocysts that implant and ones that do not, while controlling for ploidy and heterogeneity, would shed light on the pathways and genes involved in embryo implantation. We believe that uncovering the core of the transcriptomic map of an embryo that leads to live birth will open the door for novel treatment options in ART including optimization of the culturing conditions to promote critical metabolic pathways. Such an approach will potentially improve embryo prioritization for transfer as well as better define treatment factors that can influence the implantation potential of a given embryo, consequently improving the results in ART.

In conclusion, we have modified existing techniques to create a novel research platform with the possibility of rapid integration into clinical practice. With declining costs of NGS and increased accessibility of this technology, along with advances in translational methods, we are closer than ever to being able to implement a multi-omic approach when assessing the development potential of a single embryo. Further research is necessary to validate this technique and correlate it with clinical outcomes, with the goal of eventually incorporating it into the clinical setting.

## Materials and Methods

### Ethical approval

This research received approval from the University of Toronto Research Ethics Board (#30251). All patients included in this study signed an informed consent for the donation of their abnormal embryos. All experiments were performed in accordance with the relevant guidelines and regulations.

### Blastocyst selection, collection, and biopsy

A total of 25 blastocysts donated for research from a total of 21 patients were used for this study. Eight blastocysts deemed not suitable for transfer due to poor morphology were used for cell lysis and sequencing library optimization. Seventeen additional frozen blastocysts that were previously biopsied for genomic assessment and deemed unsuitable for transfer, either due to aneuploidy (n = 10) or euploid and affected by a monogenetic disease (eg. cystic fibrosis) (n = 7). These blastocysts were thawed, cultured until re-expansion occurred and their trophectoderm (TE) was re-biopsied (4–6 cells) by a clinical embryologist following the standard clinical operating procedures for TE biopsy. Supplementary Fig. S1 contains representative images of blastocysts and embryo biopsies. From each embryo two separate TE biopsies were performed where one TE biopsy was processed as a clinical sample and the other TE biopsy was utilized for evaluation of the PGT-AT method.

### Patient demographics

Patient demographic information such as age, body mass index (BMI), anti-Mullerian hormone (AMH) levels, and outcomes were collected from patients’ clinical charts. Non-identifying study numbers were assigning to all data. Patient demographics are presented in Table 1.

### Optimization of cell lysis method for TE biopsy

Eight blastocysts were used for optimizing the cell lysis step and gDNA/cDNA library preparation for next generation sequencing (NGS). Figure 1 depicts the detailed workflow of cell lysis optimization. From each of the embryos, we obtained two separate TE biopsy samples (4–6 cells) (Fig. 1). The first TE biopsy sample (TE-A) from each embryo was processed as a “clinical sample” and used as a control. TE-A was lysed and amplified using the SurePlex® WGA kit (Illumina, CA), which is the current standard of practice in PGT-A. The second TE biopsy sample (TE-B) was lysed using one of the two different lysis methods: I. SMART-seq® (Takara BioInc, CA) or II. SurePlex® kit (Illumina, CA). In lysis option I the biopsied cells were deposited into 2.5 ul of DNase/RNase free water and lysed using SMART-seq® lysis mix. For lysis option II, biopsied cells were deposited into 2.5 ul of 1xPBS buffer and lysed using cell extraction enzyme mix from SurePlex® whole genome amplification (WGA) kit. The lysates were subsequently split and independently processed for both gDNA amplification and cDNA synthesis. The resulting amplified gDNA was sequenced using Nextera XT® library preparation for NGS following the VeriSeq® protocol, described in detail below (Illumina, CA). cDNA quality was assessed using BioAnalyzer 2100 (Agilent Technologies, CA). Table 2 presents the results for chromosomal aberrations and sequencing quality metrics from VeriSeq® sequencing of gDNA obtained through SurePlex® kit using lysis with I. SMART-seq® (Takara BioInc, CA) or II. SurePlex® kit (Illumina, CA).

### Whole genome amplification (WGA), gDNA sequencing and analysis

Whole genome copy number variation (CNV) analysis was performed by whole genome low pass (0.1×) NGS using the VeriSeq® PGS Kit (Illumina, CA). Briefly, after WGA, according to manufacturer’s instructions, gDNA was tagmented and amplified. The amplified gDNA was indexed, purified using AMPure XP beads (1:1 ratio), and normalized using magnetic beads. The normalized libraries were pooled, denatured, and sequenced using a MiSeq (single-end, 1 × 36 bp). BlueFuse Multi (Illumina, CA) was used for chromosome CNV analysis and data visualization. The optimal metrics for standard clinical analysis of embryo ploidy are 500,000 reads passing filter and a sample noise score (derivative log ratio - DLR) of <0.2; 250,000 reads and DLR < 0.4 is clinically acceptable.

### Accuracy in determining the embryo ploidy status using the novel PGT-AT method

To ensure that splitting the cell lysate for simultaneous sequencing of gDNA and mRNA does not impact the ploidy status result, we thawed, expanded, and re-biopsied (4–6 cells) 17 embryos with known ploidy. In each of these cases the TE biopsy was lysed and then split: half the lysate underwent WGA and VeriSeq® sequencing and the other half of the lysate underwent cDNA synthesis and mRNA sequencing (Table 3). cDNA quality and quantity were confirmed using a BioAnalyzer 2100 (Agilent Technologies, CA). The WGA product quantity and quality were confirmed by fluorimeter (Qubit - ThermoFisher) and electrophoresis (2% w/v agarose, 100 V for 30 min), respectively.

### Low-input cDNA synthesis and amplification

cDNA was synthesized and amplified using the SMART-Seq® v4 Ultra Low Input RNA Kit for Sequencing (Takara Biosciences, CA) according to the manufacturer’s instructions. Briefly, biopsied cells were deposited into DNase/RNase free water, lysed using the optimized SurePlex® lysis method, and mRNA was selectively reverse transcribed using an oligo-dT primer (3′ SMART-Seq® CDS Primer II A). The single-stranded cDNA was amplified and purified using AMPure XP beads (Beckman Coulter, MA). cDNA size distribution was assessed using BioAnalyzer 2100 high sensitivity DNA chip (Agilent Technologies, CA).

### cDNA library preparation and RNA sequencing

One nanogram of cDNA was used as input into Nextera XT® (Illumina, CA) and libraries were generated according to manufacturer’s instructions. Briefly, cDNA was tagmented, amplified, and indexed. The indexed libraries were purified using AMPure XP beads (1:1 ratio) (Beckman Coulter, MA). Libraries were quantified by Qubit and 2100 BioAnalyzer and normalized using the KAPA Library Quantification kit (Roche, CA). Normalized libraries were pooled, denatured, and 1.2 pM was loaded onto a NextSeq High Output (300 cycle) flowcell and sequenced (paired-end, 2 × 127 bp) using a NextSeq550 (Illumina, CA).

### RNAseq bioinformatic analysis

Reads were trimmed based on read quality (Phred > 28) and aligned and quantified to hg19 using STAR^61^ (Spliced Transcripts Alignment to a Reference). Low abundant transcripts were excluded (maximum <20 in 80% of samples) and normalized using Trimmed Mean of M-values (TMM). Normalized read counts were further filtered (excluded if maximum <5 in 50% of samples) and used to perform principal component analysis (PCA) clustering and hierarchical clustering to elucidate the inter-sample variability between the samples. We conducted differential expression (DE) using DESeq2^62^ comparing euploid to all aneuploid embryos, and monosomy 16 (M16) to trisomy 16 (T16) embryos. Significantly differentially expressed genes were defined as false discovery rate (FDR) < 0.05 and fold change (FC) < −2 or FC > 2. Gene Set Enrichment Analysis (GSEA) was conducted to determine what gene pathways/gene sets are impacted by the gain or loss of chromosome 16 when compared to euploid blastocysts. This analysis was conducted in Partek Flow (version 7.0.18.0218) and the pipeline is available upon request.

### qPCR Validation of RNAseq data

Five TE lineage specific gene markers were chosen based on previous literature^9,14,21^. Validated gene-specific probe-based PrimeTime qPCR assays (IDT, IL) were used for validation of RNA-seq NGS results with the housekeeping gene, RPLP0 as the reference gene. Each PrimeTime gene-specific assay consists of two exon-spanning primers and a gene-specific fluorogenic probe labeled with FAM, and an internal and terminal non-fluorescent quencher (ZEN and Iowa Black FQ, respectively). All selected targets were assayed in duplicate using PrimeTime Gene Expression Master Mix (IDT, IL) using the following cycling conditions: polymerase activation at 95 °C for 3 min; 45 cycles of 15 s denaturation at 95 °C and 1 min annealing/extension at 60 °C. Relative fold change (ΔΔCt) method was employed to quantify gene expression. Data Analysis was performed using GraphPad Prism (version 5.02). The list of primers and probes used for validation is given in Supplementary Table S1.

## Supplementary information

Supplementary Information - Figures S1-S3 and Tables S1-S5

## Data Availability

All data generated or analyzed during this study are included in this published article (and its Supplementary Information files).
