# Supplementary material for: Towards Improving Embryo Prioritization: Parallel Next Generation Sequencing of DNA and RNA from a Single Trophectoderm Biopsy
Source: Sci Rep. 2019 Feb 27;9:2853. doi: 10.1038/s41598-019-39111-7 (PMC6393576; doi:10.1038/s41598-019-39111-7)
Supplement: Supplementary file 1 — Supplementary Information - Figures S1-S3 and Tables S1-S5 [file 41598_2019_39111_MOESM1_ESM.pdf]

TOWARDS IMPROVING EMBRYO PRIORITIZATION: PARALLEL NEXT GENERATION SEQUENCING OF DNA AND RNA FROM A SINGLE TROPHECTODERM BIOPSY

Noga Fuchs Weizman, M.D.[a,†], Brandon A. Wyse, M.Sc[a,†,*], Ran Antes, Ph.D[a], Zenon Ibarrientos, B.Sc[a], Mugundhine Sangaralingam, M.Sc[a], Gelareh Motamedi, M.Sc[a], Valeriy Kuznyetsov, Ph.D[a], Svetlana Madjunkova, M.D., Ph.D[a], and Clifford L. Librach, M.D.[a,b,c,d]

[a]CReATe Fertility Centre, Toronto, Canada; [b]Department of Obstetrics and Gynecology; [c]Department of Physiology, University of Toronto, Toronto, ON, Canada; [d]Department of Gynecology, Women's College Hospital, Toronto, ON, Canada.

a

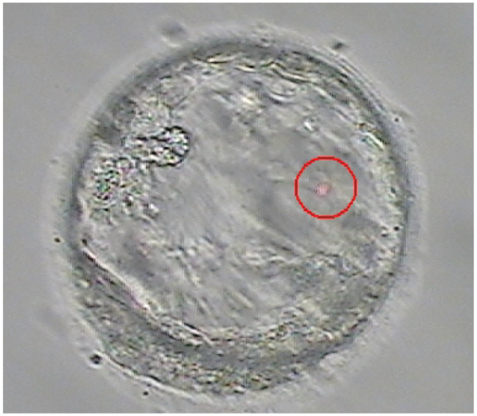

b

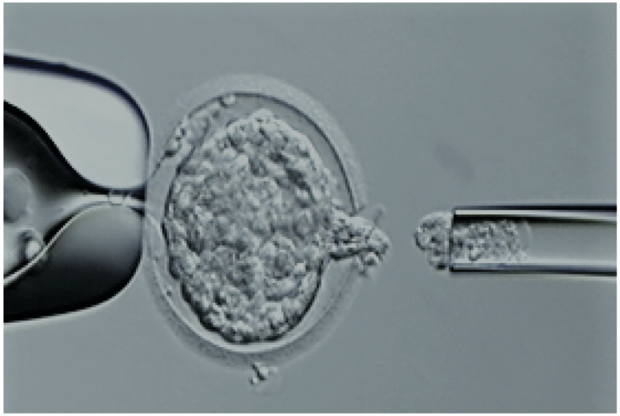

c

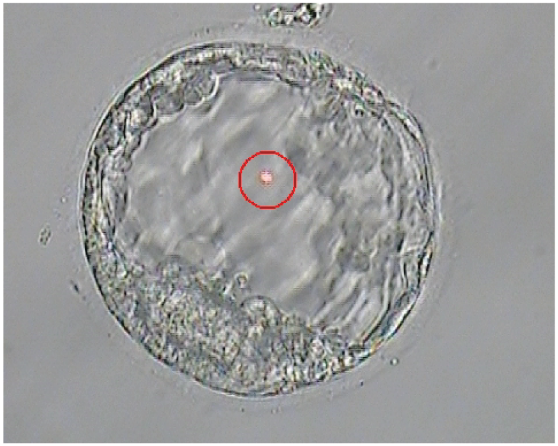

Figure S1: a) A representative image of a degenerated and late developing blastocyst deemed not suitable for transfer by an embryologist and used for lysis optimization. b) A representative image of a blastocyst being biopsied. Here the embryologist is gently aspirating 4-6 cells and removing this group of cells from the rest of the blastocyst. c) A representative image of a previously frozen blastocyst prior to re-biopsy for this study.

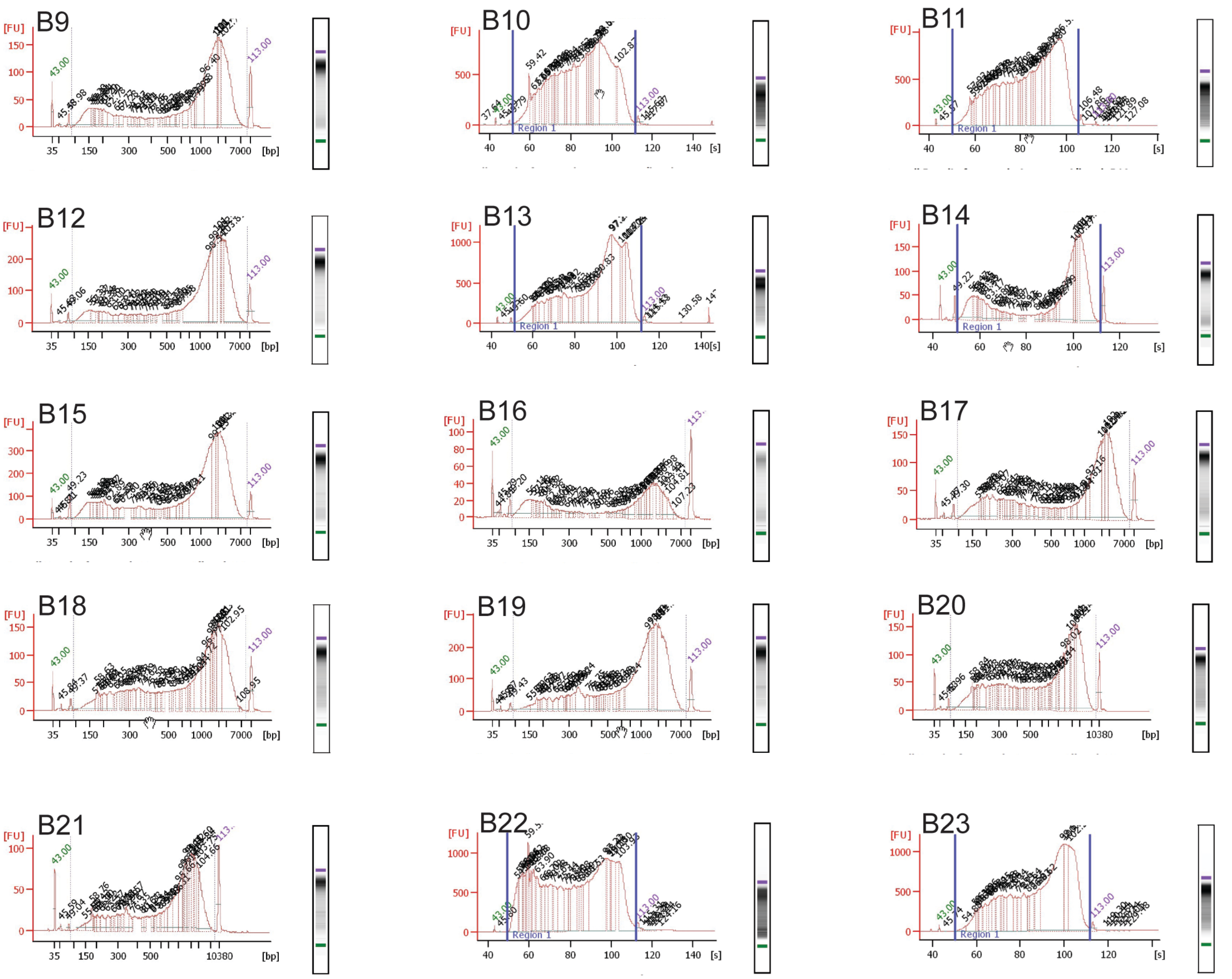

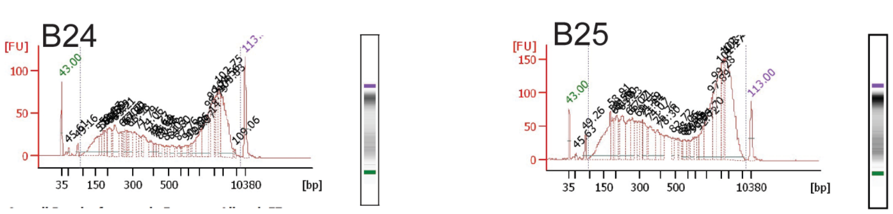

Figure S2: Bioanalyzer 2100 traces for all biopsied cells lysed using our novel PGT-AT method and cDNA synthesized using the standard SMART-seq kit. All samples yielded high-quality, full-length cDNA.

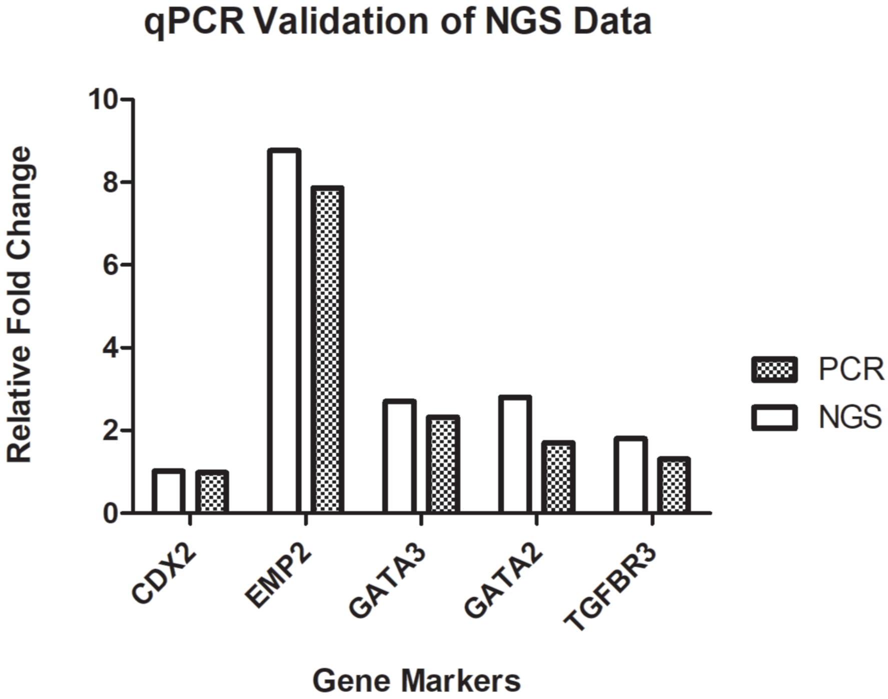

Figure S3: qPCR validation of NGS data using five selected trophectoderm specific markers. All targets had similar expression by both qPCR and NGS.

TABLE S1: LIST OF PRIMERS AND PROBES USED FOR QPCR VALIDATION OF NGS RESULTS

Target Gene		Sequence	GC (%)	Tm (50mM NaCl) (°C)
**RPLP0**	Probe	/56-FAM/CCC TGT CTT /ZEN/CCC TGG GCA TCA C/3IABkFQ/	63.64	62.12
	Primer 1	TGT CTG CTC CCA CAA TGA AAC	47.62	55.52
	Primer 2	TCG TCT TTA AAC CCT GCG TG	50.00	55.34
**CDX2**	Probe	/56-FAM/CGG CTT TCC /ZEN/TCC GGA TGG TGA T/3IABkFQ/	59.09	61.55
	Primer 1	CTT TGC TCT GCG GTT CTG A	52.63	55.38
	Primer 2	GCT GGA GAA GGA GTT TCA CTA C	50.00	55.04
**EMP2**	Probe	/56-FAM/AGC CCG GCA /ZEN/ACT AAT CCC AGT T/3IABkFQ/	54.17	60.4
	Primer 1	GAT GGA CGA GCA AAT TCC TGA	55.56	54.94
	Primer 2	GTT CAA GGG AAC AAT GAC AGA	52.38	55.29
**GATA3**	Probe	/56-FAM/CTT CGC TAC /ZEN/CCA GGT GAC CCG /3IABkFQ/	66.67	62.1
	Primer 1	GCG ACG ACT CTG CAA TTC TG	55.00	56.40
	Primer 2	CGC AAT CTG ACC GAG CA	58.82	55.42
**GATA2**	Probe	/56-FAM/TAT GCC AAC /ZEN/CCC GCT CAC GC/3IABkFQ/	65.00	63.03
	Primer 1	GCT GTG CAA CAA GTG TGG	55.56	54.86
	Primer 2	GGA CGT CTT CTT CAA TCA CCT	47.62	54.48
**TGFBR3**	Probe	/56-FAM/TGT CCA AAG /ZEN/CCC ACT TCA CCA GAT /3IABkFQ/	50.00	60.68
	Primer 1	TGG ACT TCC TCA TCT CCC AT	50.00	55.11
	Primer 2	GAT GAC ATT CCT TCA ACC CAA G	45.45	53.82

Tm – Melting temperature

TABLE S2: DIFFERENTIAL EXPRESSION OF GENES – EUPLOID VS ANEUPLOID SAMPLES

Gene ID	Total counts	FDR step up	Fold change
MAGI1	4153.22	0.00001	76.77
PSMB6	1143.10	0.00005	-46.37
FLJ31104	513.59	0.00005	364.45
KCNH5	447.28	0.00006	501.52
BAIAP2L1	1776.37	0.00007	-95.10
PTCD3	1754.95	0.00009	-71.31
CHURC1	1378.63	0.00020	-147.38
CHCHD10	684.19	0.00027	-37.85
KLF5	650.96	0.00063	-83.40
LOC107133515	792.02	0.00074	96.48
SKA1	6096.94	0.00083	71.56
PSMC6	546.34	0.00083	-80.97
GLUL	761.21	0.00083	-38.25
COX16	596.41	0.00083	-21.28
GNG5	797.67	0.00103	-87.81
MRPL4	521.53	0.00104	-68.97
CYC1	857.91	0.00107	-52.62
TRPM6	884.51	0.00130	36.82
NOP10	752.51	0.00132	-77.73
PCNA	569.64	0.00161	-63.04
RPGRIP1L	1358.34	0.00195	72.25
PTTG1	625.54	0.00225	-93.20
SLC31A1	1672.98	0.00225	14.89
PATJ	2018.34	0.00225	18.47
RNPEP	590.84	0.00227	20.70
NEMP1	369.85	0.00232	110.87
DARS	498.60	0.00255	-26.47
AKAP12	5638.86	0.00255	5.58
C8orf33	469.59	0.00255	-70.16
TRAPPC6B	1800.42	0.00255	69.17
RRM2	1292.01	0.00274	-38.60
LAMTOR2	339.23	0.00288	-61.46
GLRX5	591.22	0.00288	-35.22
ACADVL	288.45	0.00288	-62.05
GRK6	425.92	0.00306	-89.08
ARF4	437.11	0.00312	-67.76
H2AFX	409.89	0.00328	-30.51
CD151	303.41	0.00335	-47.08
TMEM108-AS1	1533.00	0.00348	127.61
RAB7A	629.65	0.00354	-22.61
SNHG17	385.29	0.00354	-49.68
KIAA0754	1840.39	0.00354	46.47
CEBPA	359.58	0.00354	-57.57
DDX39A	824.94	0.00354	-25.92
ANAPC7	1047.12	0.00354	15.66

GTF2H5	320.03	0.00354	-64.09
TRIM28	2489.78	0.00382	-16.55
ERRFI1	344.52	0.00393	-55.12
INCENP	1026.02	0.00393	13.74
C2orf48	945.97	0.00393	101.90
MDH1	380.86	0.00404	-47.21
MACF1	3526.35	0.00408	13.71
HK2	1705.02	0.00408	-30.03
MAP2K2	456.00	0.00408	-24.13
POLR2I	321.28	0.00415	-53.97
WDR75	441.00	0.00421	-51.69
ANGEL1	2489.78	0.00481	49.67
MRPL38	540.65	0.00492	-12.97
RPAP3	513.69	0.00492	-21.74
ZNF502	1037.90	0.00502	87.29
SENP5	3308.50	0.00502	14.53
KLF7	6436.37	0.00502	71.64
KIAA1549	15345.05	0.00508	34.17
JUP	685.82	0.00508	-14.72
GABARAPL2	334.71	0.00575	-54.93
ZNF652	3123.16	0.00604	40.34
UBR4	1351.78	0.00606	5.94
MRPL22	906.70	0.00633	-37.60
COPS9	473.82	0.00637	-35.08
ZNF543	1551.02	0.00677	45.31
C4orf3	361.23	0.00677	-33.75
SYNGR2	611.09	0.00677	-44.14
TMEM258	531.79	0.00677	-25.09
MPP5	1486.97	0.00677	16.96
JAGN1	604.42	0.00677	-30.71
ATP6V1F	418.08	0.00682	-45.63
KXD1	295.39	0.00682	-45.40
DVL1	912.61	0.00686	-16.21
TXNL4B	1340.32	0.00713	51.53
SKP1	8823.78	0.00715	16.54
KIAA1143	299.35	0.00715	-25.79
SNAPC4	233.10	0.00736	-34.83
WAC-AS1	987.52	0.00745	20.00
NCOR2	255.86	0.00778	-26.51
SHKBP1	168.05	0.00784	-52.77
ZBTB44	606.39	0.00784	-39.84
DUS3L	215.31	0.00784	-42.80
MRPL15	581.34	0.00784	-18.25
KALRN	2020.84	0.00784	26.35
MRPS6	237.68	0.00784	-48.29
ZNF578	838.57	0.00784	37.12
ZNF417	1709.36	0.00784	29.81

PPP1R14B	806.67	0.00793	-10.55
RPLP0P2	903.52	0.00801	27.54
DNAJC5	190.46	0.00802	-34.78
EIF3M	830.00	0.00814	-17.53
PRODH	518.86	0.00831	-19.43
NDUFAF4	302.61	0.00832	-59.23
LIMCH1	1207.19	0.00855	82.95
RANGAP1	6713.95	0.00862	9.31
RAB18	468.51	0.00862	-33.71
PGM2L1	1542.64	0.00869	22.27
PKP4	851.23	0.00871	27.81
COX8A	758.32	0.00871	-25.89
ATP5D	1722.12	0.00871	-8.34
DCP2	3222.36	0.00871	19.98
ZNF431	1600.97	0.00871	31.07
CAMK2G	328.72	0.00897	-36.01
ALG2	1294.38	0.00899	-34.88
RPP21	172.78	0.00907	-42.72
FAM49B	319.22	0.00920	-39.97
ABHD11	125.50	0.00920	-38.61
PRDX2	496.46	0.00920	-26.68
MTPAP	3778.57	0.00923	20.21
RPUSD1	252.88	0.00923	-42.83
ZC3H18	726.41	0.00923	7.94
LRRC47	423.15	0.00924	-20.61
ABHD5	1089.98	0.00942	30.43
PPP4R3B	259.38	0.00942	-35.28
SNX3	352.98	0.00965	-22.54
MDN1	5541.43	0.00967	5.44
LOC646762	3307.23	0.00967	79.61
FAT3	2254.00	0.00967	50.13
TTC37	337.14	0.00978	-25.23
CS	378.92	0.00996	-16.12
EIF4EBP2	495.85	0.00997	-15.69
HEBP2	590.77	0.00998	-17.01
TTLL3	1077.42	0.01046	-34.31
UBE2E3	384.46	0.01059	-49.98
UBE2A	260.03	0.01067	-41.71
PKP2	444.15	0.01067	-19.92
ZNF789	1009.75	0.01085	61.48
EXOSC8	213.65	0.01095	-39.34
TIMM23	808.69	0.01095	-35.94
ENTPD7	6708.50	0.01132	66.82
ZNF286B	1598.21	0.01172	23.89
CCDC85C	381.63	0.01172	-35.03
TFG	407.11	0.01178	-30.98
GLOD4	255.83	0.01178	-17.33

GNB1	577.55	0.01206	-10.64
SLK	1361.88	0.01212	-7.48
NUTF2	269.99	0.01227	-16.85
PURA	249.72	0.01238	-24.66
CITED4	698.26	0.01238	-13.28
KRTCAP2	149.90	0.01239	-37.43
LRRFIP2	160.05	0.01244	-43.76
C19orf53	469.86	0.01244	-19.60
PALM2	2698.20	0.01272	49.43
FAM184B	1184.73	0.01272	40.56
RNF11	241.21	0.01272	-46.99
DIXDC1	2197.90	0.01282	45.89
SH3GLB2	315.37	0.01282	-23.84
ZBTB40	8811.01	0.01285	25.75
PYGO1	4148.84	0.01285	66.95
RETREG3	1536.02	0.01332	34.76
CAPNS1	361.15	0.01340	-33.71
RUNDC1	1752.47	0.01374	47.74
NBEAL2	193.60	0.01374	-39.26
RPL15	3823.16	0.01388	-6.92
ZFP91	495.50	0.01398	-20.06
VPS37C	159.32	0.01412	-40.42
L2HGDH	1092.05	0.01454	26.04
ZNF714	1539.08	0.01479	18.44
ERCC1	1744.93	0.01479	17.33
NOL9	1789.81	0.01496	5.01
DIMT1	1425.27	0.01496	25.29
RAB35	254.29	0.01496	-18.06
CCDC186	1349.92	0.01496	-22.58
ASL	521.18	0.01496	-30.74
NUBP2	404.97	0.01496	-33.15
ANP32A	1572.84	0.01496	9.02
ZAK	3698.63	0.01496	42.31
BDP1	5314.54	0.01496	4.00
CNGA3	2424.35	0.01496	50.46
NDOR1	249.29	0.01496	-18.93
CTDSP1	183.93	0.01496	-19.84
DDX41	348.36	0.01499	-17.48
MAT2A	237.89	0.01521	-33.85
PDCD5	694.51	0.01521	-14.89
MTCH1	748.18	0.01547	-29.52
TRUB2	179.88	0.01556	-32.44
EMC4	583.66	0.01556	-29.42
NDUFB3	788.34	0.01556	-30.65
GOLPH3	306.88	0.01627	-34.07
PPP1R16A	186.94	0.01629	-36.92
ETFA	386.41	0.01629	-22.42

SLC52A2	210.32	0.01629	-33.37
RAB11A	9254.08	0.01695	16.27
CSNK1D	590.36	0.01810	-16.03
PSMD3	216.30	0.01816	-13.50
SMAGP	227.68	0.01851	-33.95
CLIC4	361.27	0.01859	-16.77
RPAIN	177.68	0.01859	-23.75
SCIMP	3084.17	0.01859	33.03
ZNF496	1736.11	0.01859	38.16
PCMTD1	703.76	0.01862	24.82
CAMLG	172.98	0.01862	-34.24
FOXN3	2213.39	0.01862	13.47
MED28	197.36	0.01869	-30.99
ZCCHC2	2056.32	0.01869	33.47
MNAT1	287.21	0.01869	-33.43
BOLA3	275.90	0.01903	-36.45
PIAS2	5693.19	0.01903	15.64
RPS13	5196.18	0.01903	-7.29
RPL39L	223.73	0.01903	-43.34
WDR18	209.12	0.01921	-28.42
AKIRIN2	137.43	0.01924	-30.15
ZNHIT6	1923.37	0.01924	13.22
MZT2B	271.30	0.01924	-30.44
ARMC1	596.23	0.01924	-21.70
IRF2BP2	80.18	0.01924	-27.45
UNC13B	219.65	0.01937	-33.91
ARMT1	204.62	0.01943	-32.60
CHAF1A	1305.21	0.01943	5.41
PRELID3B	193.77	0.01943	-29.02
TBL2	447.41	0.01947	-29.40
GLB1	162.33	0.01959	-30.81
PER2	293.36	0.01959	-27.20
GAS5	519.89	0.01983	-10.92
PGK1	1566.72	0.01984	-7.71
HPDL	129.94	0.02005	-33.50
TADA2B	1118.84	0.02033	23.37
ESRRA	332.30	0.02033	-21.84
CRNKL1	536.05	0.02033	-13.87
NDUFA1	2899.65	0.02033	-12.91
METTL1	198.84	0.02038	-26.43
SMIM26	125.85	0.02040	-33.83
BRCC3	1063.62	0.02046	-19.71
MARVELD2	806.58	0.02046	29.50
AKR1A1	393.64	0.02046	-24.36
ARL4C	276.95	0.02048	-28.09
ITPA	226.75	0.02049	-30.17
7-Sep	917.19	0.02049	-11.36

ATP5G1	265.94	0.02054	-33.21
ELK4	737.64	0.02073	26.06
RYR3	750.23	0.02113	21.20
DYNLRB1	206.63	0.02124	-23.05
SAPCD2	1831.32	0.02143	-8.60
DNM2	282.38	0.02143	-10.05
PNKP	157.96	0.02161	-25.46
GTSF1	153.49	0.02182	-23.25
RN7SL832P	322.36	0.02182	38.85
WASF2	783.06	0.02182	-8.73
ZNF736	1173.03	0.02188	35.25
MEX3C	4152.88	0.02204	-23.55
SLX4IP	2313.82	0.02226	50.03
CDK5RAP1	217.21	0.02248	-19.11
OS9	1065.55	0.02255	11.82
LINC00470	2131.00	0.02264	27.02
COPE	237.46	0.02264	-16.68
NIFK	648.31	0.02271	-11.75
GNAI2	140.29	0.02271	-25.82
FAM162A	261.11	0.02280	-35.44
TSNAX	385.64	0.02292	-28.84
PPP1R15B	3449.51	0.02313	16.79
LINC00987	993.88	0.02349	3.76
PLEKHH3	192.07	0.02349	-27.95
LOC284454	1132.42	0.02349	24.27
LIMD1	1588.68	0.02367	14.35
YY1AP1	313.03	0.02398	-35.58
SENP3	196.83	0.02414	-30.03
UROD	166.65	0.02423	-27.34
THOC7	136.73	0.02423	-28.09
FDXR	154.45	0.02425	-33.10
U2AF2	688.00	0.02425	7.95
IGF1	560.27	0.02425	35.07
ZNF611	1113.16	0.02428	11.63
RPS6KA5	1647.71	0.02428	7.72
SELENOH	421.19	0.02441	-14.63
CHST15	611.97	0.02466	13.63
BUD31	350.35	0.02474	-17.36
RAB1B	165.62	0.02475	-15.56
RYK	95.53	0.02475	-25.35
PSMB3	863.79	0.02475	-8.55
FYCO1	1032.02	0.02480	-26.37
MYEF2	2726.94	0.02480	31.27
ZCCHC10	182.99	0.02481	-16.99
LTA4H	143.07	0.02501	-30.21
MAEA	643.94	0.02501	-18.53
ABHD12	211.72	0.02508	-12.57

CCNJ	282.39	0.02508	25.76
ADAR	2117.70	0.02526	8.27
CYB5B	281.52	0.02527	-26.40
GSTP1	3383.91	0.02527	-7.20
ARFGAP1	366.86	0.02527	-11.09
TMEM237	841.86	0.02527	20.64
KIAA0930	2489.76	0.02527	17.48
C1D	95.61	0.02527	-34.10
ANKRD54	2823.63	0.02527	24.93
CDX1	306.79	0.02531	-33.82
POMGNT1	540.66	0.02569	-14.39
SLC5A3	1897.25	0.02569	25.92
PIP4K2C	106.11	0.02591	-30.55
CXorf23	145.38	0.02591	-28.99
MAGOH	286.10	0.02591	-27.88
SSR1	2818.97	0.02591	11.93
SETD6	216.58	0.02613	-29.24
CDC27	2268.80	0.02619	6.95
PARP2	215.95	0.02622	-21.09
KAT2A	231.49	0.02632	-27.16
SPC25	118.51	0.02635	-27.49
ZNF536	1925.81	0.02635	29.22
SERF2	2868.70	0.02643	-6.99
GLUD1	518.32	0.02656	-16.93
FBLIM1	1531.89	0.02656	23.87
SMIM15	151.17	0.02656	-27.56
VPS25	221.42	0.02656	-23.27
SLC25A33	99.39	0.02656	-23.67
DAND5	4256.78	0.02684	29.25
ENTPD1	2030.35	0.02684	9.40
UBE2B	283.76	0.02684	-24.70
SYPL1	277.88	0.02723	-22.60
ORC2	4566.08	0.02723	21.20
IPO7	964.55	0.02723	-7.79
PCBP1	1343.70	0.02723	-9.77
DDX6	3111.86	0.02723	13.53
EIF3H	1212.82	0.02723	-10.78
SLC9A3R1	525.19	0.02758	-9.66
ARF3	266.16	0.02760	-23.75
RAD54B	499.38	0.02760	15.71
DR1	191.39	0.02764	-32.46
DHPS	270.58	0.02801	-19.28
PPP1CC	853.46	0.02803	-7.83
BCR	461.47	0.02803	-6.96
MIR600HG	180.53	0.02803	-33.91
PAX5	656.45	0.02830	30.74
CHD9	2025.98	0.02884	28.76

OTUD3	131.74	0.02905	-22.73
RNASEH2C	506.78	0.02911	-16.32
RNF111	238.25	0.02915	-16.72
ATP11B	157.55	0.02948	-26.77
VPS29	228.58	0.02987	-25.53
IPO4	344.63	0.02997	-14.06
DLGAP4	98.85	0.02997	-24.70
CTPS1	209.28	0.02997	-16.76
GAPDH	10706.37	0.02997	-5.61
GTF2F2	263.88	0.02997	-30.12
ERH	335.13	0.02997	-11.53
DPP9	123.24	0.02997	-23.70
NAXE	202.85	0.03015	-24.55
VKORC1	171.65	0.03021	-28.71
RAB25	258.22	0.03021	-17.70
KANSL2	132.91	0.03030	-27.74
ZYG11A	1900.70	0.03030	21.79
C5orf22	533.67	0.03032	-26.66
ATG16L1	197.54	0.03032	-33.99
ZMYM5	1227.73	0.03032	32.25
CLCN3	587.21	0.03032	9.01
ZNF706	232.13	0.03055	-13.00
PDCD6	359.76	0.03109	-15.11
COX7B	1607.78	0.03133	-9.66
TRAK2	1223.02	0.03133	16.28
CDK16	129.17	0.03164	-27.35
SLC25A5	2822.48	0.03198	-13.31
GRAMD4	1758.25	0.03227	21.44
KCNK6	1824.43	0.03240	18.86
ARHGEF18	164.33	0.03251	-25.10
MTDH	6894.20	0.03273	5.96
HELZ	5975.24	0.03275	14.31
TRABD	230.25	0.03297	-20.39
RAB30	1904.90	0.03313	39.74
NCL	42014.21	0.03330	3.75
CDK6	1079.39	0.03330	-12.01
BLZF1	689.13	0.03356	17.10
TPP2	3175.05	0.03356	12.83
PPP1R14A	649.36	0.03358	-11.57
TMEM199	81.44	0.03366	-27.05
LOC644936	1333.58	0.03366	13.43
PDIA4	1495.40	0.03366	9.26
VHL	2735.89	0.03366	5.35
TTL	163.09	0.03366	-30.04
NUS1	216.62	0.03368	-11.61
TMEM39A	1690.49	0.03387	25.92
SCGB3A2	383.41	0.03395	-25.88

FAM161A	609.48	0.03395	26.93
ARFRP1	291.32	0.03395	-13.09
TOLLIP	92.19	0.03427	-25.85
POLG	597.89	0.03427	10.54
NDUFA12	1741.67	0.03427	-19.31
FIS1	147.78	0.03428	-13.84
PNRC2	145.31	0.03442	-19.87
RAB1A	690.97	0.03476	-10.01
NDUFB6	465.11	0.03494	-10.87
UBIAD1	2633.96	0.03507	10.68
DNPH1	82.61	0.03508	-24.99
CBX5	2815.37	0.03547	12.76
PIN1	319.69	0.03547	-21.48
CERS2	136.59	0.03547	-15.31
UBE2J2	1031.51	0.03547	-13.88
PTCD1	959.65	0.03552	11.55
L3MBTL3	316.95	0.03558	-24.30
ACOT13	2303.95	0.03567	-34.95
CBX1	644.94	0.03619	-8.55
CORO1C	442.98	0.03672	-5.36
ZNF91	432.52	0.03673	12.08
RPP25	126.57	0.03698	-32.40
RBPMS2	1344.66	0.03737	-13.91
XAB2	124.80	0.03737	-18.86
LINC00537	4680.33	0.03737	7.47
RPF1	163.08	0.03737	-19.12
LGR6	2951.85	0.03737	29.16
FADS1	1612.73	0.03739	10.89
SMG7	280.12	0.03759	-8.87
ROCK2	1149.68	0.03768	10.41
VASP	326.38	0.03768	-23.09
MUM1	728.36	0.03768	9.23
CLPP	429.21	0.03785	-11.99
PRAG1	426.90	0.03791	-25.80
REXO2	189.52	0.03791	-22.69
ATP8B1	1566.79	0.03825	7.22
TPRA1	101.72	0.03839	-19.25
THAP3	268.63	0.03839	-12.79
ATP1B3	3285.29	0.03839	5.22
ESRG	116.07	0.03847	-14.50
CENPN	480.90	0.03873	-14.47
HNRNPM	6367.99	0.03874	2.54
TAPBP	1017.67	0.03874	25.38
ANAPC5	268.86	0.03874	-14.85
ANAPC10	89.02	0.03894	-27.97
DYNLL1	256.02	0.03894	-15.17
ATXN3	2141.64	0.03895	20.89

HSD17B7	124.42	0.03900	-24.66
SLC25A19	104.96	0.03911	-25.49
ACER3	1140.77	0.03913	10.01
UIMC1	896.56	0.03925	13.72
PSMD6	393.92	0.03972	-10.62
RAB5A	143.65	0.03972	-21.85
PPAT	427.31	0.03973	-11.67
TEAD3	575.20	0.03973	14.55
KANSL3	341.73	0.03983	14.05
GOT1	93.33	0.04006	-14.95
SMARCD1	136.84	0.04006	-27.30
SMYD5	168.72	0.04006	-24.55
UBAC2	460.56	0.04006	-23.47
PNPLA8	2547.26	0.04006	16.23
GALM	1032.29	0.04059	17.60
ZC3H12B	3902.14	0.04059	22.41
TALDO1	952.96	0.04059	-8.50
TFDP1	192.33	0.04059	-18.68
RAB2B	586.52	0.04059	20.17
PRPF40A	1621.74	0.04086	-4.87
CDC16	140.44	0.04087	-14.02
SRP14-AS1	467.67	0.04093	-28.64
NDUFA2	980.35	0.04096	-11.61
SH3GL1	364.96	0.04113	-14.26
DIP2A	1256.58	0.04113	9.57
CRELD2	301.85	0.04135	-20.86
ARHGEF12	346.82	0.04139	-16.22
ZNF276	1405.36	0.04149	18.77
POLR2H	474.33	0.04167	-11.64
FRYL	2811.66	0.04168	8.53
TRAF4	203.34	0.04170	-8.53
CUX1	1541.63	0.04180	16.34
C20orf27	130.69	0.04180	-18.11
RTKN	81.53	0.04180	-22.11
RMND1	1698.29	0.04180	15.17
SFXN1	797.49	0.04203	-14.73
PGLS	139.69	0.04234	-29.72
FLII	571.53	0.04237	-17.28
EIF4EBP1	383.04	0.04237	-10.60
PI4K2A	410.15	0.04240	-16.32
POLR2J	389.29	0.04240	-12.94
MND1	257.52	0.04242	-19.68
H1FX	339.81	0.04272	-10.61
MRPS7	592.20	0.04273	-8.31
MRPL48	135.92	0.04273	-25.53
KLHL24	5531.95	0.04273	18.15
TRMT10A	359.91	0.04273	-19.63

ATXN2L	115.39	0.04273	-16.58
USF2	215.31	0.04273	-10.61
CDC6	224.18	0.04273	-14.76
SS18L2	153.44	0.04273	-20.91
DIABLO	289.59	0.04273	-11.50
PHF6	1636.72	0.04273	16.60
CDS2	3872.84	0.04273	11.69
TUBB2A	166.68	0.04273	-23.72
UBA3	539.21	0.04273	-10.92
ZNF557	461.70	0.04273	15.78
ANKIB1	194.83	0.04273	-10.28
ZNF644	204.21	0.04273	-20.45
EID1	281.03	0.04293	-20.77
PFN1	2109.19	0.04302	-4.69
RAD17	138.88	0.04360	-23.69
C22orf46	1239.83	0.04365	28.24
TBC1D7	2200.31	0.04376	20.75
PDLIM1	564.09	0.04377	-12.22
FOXR1	113.88	0.04438	-21.27
ZNF468	1551.73	0.04444	13.70
PCYT2	395.15	0.04468	-9.21
MTRF1L	198.42	0.04477	10.20
ARL15	560.45	0.04487	17.70
MDM4	5186.50	0.04491	7.30
SUMO2	488.53	0.04491	-6.95
COPS2	354.32	0.04491	-12.13
MRI1	426.78	0.04491	-19.02
OAT	598.56	0.04491	-13.04
UQCR11	1841.76	0.04491	-7.76
LARP6	1475.04	0.04491	-19.34
HPGD	353.89	0.04491	-14.73
DOK4	376.33	0.04491	18.91
RAVER1	91.11	0.04491	-19.29
UBE2D2	3874.69	0.04491	-15.28
ARPC4-TTLL3	523.59	0.04491	-28.62
MLLT10	178.14	0.04491	-11.48
RABGGTB	847.67	0.04491	-9.44
SBDS	452.63	0.04521	-13.77
PPP1R15A	282.36	0.04540	-15.32
EMP2	2809.89	0.04553	8.76
CEBPZOS	475.89	0.04601	-8.54
VCX3A	785.82	0.04601	18.45
SIPA1L2	517.63	0.04626	13.66
COASY	1509.02	0.04626	7.48
MPDU1	128.82	0.04626	-16.10
FRG1	324.21	0.04626	-10.94
PPP6C	205.65	0.04626	-21.09

TRAPPC3	105.50	0.04626	-23.43
IGF1R	1083.65	0.04643	14.01
CDK4	452.03	0.04643	-21.78
SBDSP1	208.40	0.04661	-17.98
NDUFB11	594.29	0.04681	-7.20
HSP90AA1	76526.23	0.04724	2.88
SLC24A1	2101.24	0.04728	30.82
DERL2	6347.25	0.04729	18.19
STOML2	928.80	0.04741	-9.05
SLC30A7	1772.04	0.04747	14.75
PPP2R5C	362.79	0.04747	-15.29
RAD51C	212.39	0.04793	-23.03
RPL18A	731.94	0.04793	-4.28
XIAP	3838.07	0.04814	9.88
LAMC2	3139.78	0.04837	11.72
UPF2	2866.46	0.04849	3.67
SORBS1	552.79	0.04849	-17.42
ALDH16A1	275.24	0.04981	-18.52
CRIP1	1557.30	0.04985	-7.12

TABLE S3: DIFFERENTIAL EXPRESSION OF GENES – EUPLOID VS MONOSOMY 16 SAMPLES

Gene ID	Total counts	FDR step up	Fold change
FAM184B	406	0.00144	677.93
ERVK13-1	3270	0.00355	65.48
PAX5	372	0.00355	232.04
PCBP1	465	0.00355	-70.87
STYX	1810	0.00355	114.18
CHURC1	625	0.00472	-289.62
PTCD3	403	0.00639	-84.51
ANGEL1	1320	0.00767	192.60
C8orf33	194	0.00767	-199.54
EMP2	1190	0.00767	40.30
PLXNA2	1420	0.00830	88.50
NAXD	179	0.01420	-121.29
CEP350	5080	0.01630	40.65
CADM1	3620	0.01630	63.30
CNGA3	1470	0.01630	94.11
ZNF417	824	0.01630	43.94
NOP10	190	0.01630	-126.19
MAGI1	2380	0.01630	43.78
NDUFA6	932	0.01630	-24.34
MARVELD3	419	0.01630	75.72
RNPEP	268	0.01630	49.93
CEBPA	117	0.01630	-140.78
PCNA	123	0.01930	-137.79
FHL2	110	0.01930	-80.58
PSMC6	160	0.01930	-116.17
NT5C3B	306	0.02020	-66.18
TRPM6	430	0.02020	50.34
GTF2H5	144	0.02020	-137.05
ASXL1	98	0.02050	-74.35
ZNF543	658	0.02050	63.63
ADA	98	0.02050	-120.67
RPS23	2210	0.02160	-10.31
CYC1	195	0.02160	-88.48
GNG5	186	0.02160	-127.67
TAF10	205	0.02170	-82.15
MRPL4	116	0.02230	-107.44
ZNF546	1840	0.02420	81.76
MTCH1	338	0.02420	-96.32
RPS18	2500	0.02530	-17.88
GDF15	475	0.02530	-53.59
HBS1L	261	0.02530	-77.75
ARFRP1	132	0.02650	-31.38
LSM3	208	0.02800	-32.27
ATP5G1	62	0.03060	-86.39
RPL10	999	0.03060	-15.53
MICAL2	284	0.03060	-67.74
UBE2V2	551	0.03060	32.18

FAM162A	119	0.03060	-103.64
RPGRIP1L	577	0.03060	55.16
WDR75	121	0.03090	-98.21
KLF5	150	0.03140	-95.13
DNAJC5	39	0.03260	-66.53
PHF6	933	0.03270	60.21
LAMTOR2	111	0.03270	-97.72
TMEM258	136	0.03270	-65.87
INCENP	531	0.03280	16.22
EIF4H	223	0.03280	-58.36
LSM7	236	0.03600	-26.55
RPAP3	219	0.03620	-37.62
RPUSD1	40	0.03620	-65.89
RAD17	48	0.03620	-68.55
GNL3L	795	0.03680	12.70
POLR2H	132	0.03680	-46.94
KIF1BP	22	0.03680	-46.94
TPP2	1770	0.03870	34.39
PSMB6	199	0.03870	-41.25
PURA	72	0.03890	-54.48
ZNF652	1450	0.03890	52.78
ZNF536	922	0.03900	45.84
FTL	890	0.03930	-25.68
CHCHD10	182	0.03930	-39.89
TIMM9	55	0.03990	-69.38
RBFOX2	36	0.04180	-55.61
SNAPC4	44	0.04180	-59.95
SNHG17	71	0.04180	-73.58
MACF1	1900	0.04180	15.01
CDK5RAP1	37	0.04180	-33.46
RPP21	55	0.04180	-66.29
TMEM199	34	0.04180	-52.53
EFNB2	363	0.04180	51.34
CDYL	121	0.04180	-50.35
AKAP12	2320	0.04180	6.10
RPL30	1140	0.04180	-15.21
GRK6	224	0.04180	-54.45
POLR2I	98	0.04180	-72.32
MRPS6	77	0.04280	-70.16
7-Sep	243	0.04280	-26.15
ZC3H18	385	0.04330	16.17
CHST15	337	0.04340	18.98
ERRFI1	74	0.04340	-69.18
KCNK6	891	0.04340	29.76
NCOR2	86	0.04340	-60.29
H2AFX	131	0.04340	-45.64
COX8A	274	0.04340	-27.85
CLINT1	246	0.04340	-48.26
ARF4	135	0.04340	-69.53

MSMO1	636	0.04340	-21.47
PI4KA	177	0.04460	-33.98
C19orf53	125	0.04460	-37.49
MTPAP	2080	0.04540	32.08
CTDSP1	48	0.04640	-41.00
NEXN	189	0.04640	26.15
PKP2	120	0.04680	-28.82
MAP2K2	152	0.04680	-35.45
SENP5	1660	0.04760	14.58
NUP50-AS1	59	0.04760	-65.34
BROX	49	0.04770	-57.82
AKIRIN2	47	0.04770	-56.60
RBM33	1050	0.04770	18.63
COPS9	116	0.04770	-41.53
COX16	162	0.04770	-18.71
PPP1R14B	248	0.04770	-21.96
LRRC47	209	0.04770	-27.50
TRAF4	48	0.04770	-29.19
PLEKHH3	85	0.04770	-64.42
SCFD1	128	0.04770	-38.00
SCIMP	1660	0.04770	35.85
CRELD2	53	0.04770	-56.00
CKS2	719	0.04770	-26.17
FOXR1	39	0.04770	-49.53
FBXO46	890	0.04790	27.33
PIN1	27	0.04800	-41.30
GET4	462	0.04800	-16.33
CDK16	51	0.04800	-54.33
NDUFB7	892	0.04800	-14.78
ASAP2	147	0.04860	-42.64
SLC5A6	276	0.04860	-13.10
LOC284454	585	0.04860	39.53
BRD7	190	0.05000	10.97

TABLE S4: DIFFERENTIAL EXPRESSION OF GENES – EUPLOID VS TRISOMY 16 SAMPLES

Gene ID	Total counts	FDR step up	Fold change
RNR2	1090024.45	0.000000	8.92
RNR1	133534.72	0.000001	9.35
PALM2	1316.23	0.000001	2684.49
CELF2	2498.25	0.000002	1435.71
TMEM108-AS1	836.99	0.000004	1864.01
ZNF614	6737.30	0.000018	1171.13
P2RX7	461.38	0.000018	606.32
CNGA3	1698.92	0.000025	1353.11
SOCS2	1084.06	0.000237	942.13
AFF1	826.16	0.000237	703.23
ZNF431	905.46	0.000273	378.57
FAM227A	507.01	0.000273	371.76
LGR6	1873.76	0.000367	838.67
ERCC1	1107.06	0.000367	122.64
SVEP1	203.00	0.000374	310.86
STAR	363.79	0.000395	705.10
ZC3H12C	2524.88	0.000598	706.20
LINC01977	606.10	0.000598	467.62
HSD3BP4	1519.70	0.000598	609.50
KCNH5	256.41	0.000697	587.73
PLEKHA5	278.07	0.000697	40.63
PDGFD	377.13	0.001136	552.79
ZNF251	452.80	0.001209	544.56
RNF144B	378.30	0.001212	532.67
KIAA0754	524.91	0.001242	300.84
RIN2	370.56	0.001242	519.70
TNFAIP8	2482.80	0.001242	228.16
PHLDA1	1112.11	0.001345	180.33
ZIK1	421.87	0.001395	270.79
MTRNR2L2	9391.27	0.001426	8.55
LRRN3	308.20	0.001636	336.26
IGF1	294.72	0.001918	445.83
PSMB6	185.37	0.002684	-170.01
MPP5	845.48	0.003187	32.47
ZAK	2478.19	0.003279	262.17
IGF2BP2-AS1	638.41	0.003502	309.02
TRIM52-AS1	319.16	0.004091	244.74
BAIAP2L1	156.14	0.004845	-157.15
LOC101926935	483.32	0.005051	86.11
RAD54B	239.54	0.007719	105.92
GPR155	590.82	0.008324	81.20
DCAF4L1	4969.50	0.008324	79.21
RNF138P1	1331.08	0.009810	210.63
RAB2B	335.28	0.010800	199.03
ZFP14	1250.09	0.011328	106.69
GLRX5	144.30	0.011497	-140.81
LTBP1	590.38	0.011762	107.08
PDZD2	1148.15	0.012014	259.15
SH3PXD2A	1927.42	0.012322	195.89
ZNF578	268.91	0.012322	71.58
ADGRV1	167.90	0.012484	96.32
UBE2E3	107.47	0.012759	-139.04
INA	98.33	0.012759	-150.00

KALRN	1019.50	0.012759	45.10
ZNF543	727.58	0.012949	81.23
NOS1AP	664.50	0.013065	152.56
BRCC3	103.30	0.013727	-106.38
GNG5	131.34	0.013738	-156.11
PDLIM1	148.11	0.015583	-58.88
YY1AP1	71.81	0.016083	-119.84
PTTG1	67.34	0.016724	-112.29
PRR13	529.74	0.016724	-66.29
KCNK6	964.25	0.016724	38.85
GABARAPL2	68.08	0.016724	-116.14
RNF11	77.73	0.016724	-124.66
SYNGR2	65.80	0.016724	-113.74
MTRNR2L3	803.92	0.016724	8.56
CCDC85C	71.62	0.016724	-119.15
MUM1	376.32	0.016724	35.74
ERRFI1	72.07	0.016724	-119.22
NUTF2	95.13	0.016724	-92.62
SETD6	71.64	0.016724	-117.59
MRPS26	160.50	0.016724	-95.74
ZNF589	60.95	0.016724	-99.95
KLF5	80.81	0.016724	-125.35
KLHL28	635.72	0.016724	103.60
LINC01754	178.30	0.016724	181.08
PGLS	54.98	0.016724	-96.45
NUBP2	88.10	0.016724	-130.16
CDIPT	51.63	0.016724	-92.10
CLIC4	87.65	0.016724	-83.31
OLR1	53.73	0.016724	-99.30
SPG11	172.91	0.016724	24.95
SPICE1	783.43	0.016724	74.16
GINS2	63.63	0.016724	-109.18
KAT2A	51.09	0.016724	-95.95
SNHG17	55.44	0.016724	-97.82
SLC31A1	927.47	0.016724	11.01
PAX5	422.69	0.016724	57.78
MED28	49.36	0.016724	-93.95
ZNF267	46.30	0.016724	-83.83
MTRNR2L8	3799.81	0.016724	7.11
ARL4C	44.21	0.016724	-86.31
PPP1R16A	41.56	0.016724	-82.25
UBE2A	46.20	0.016724	-87.52
CDC27	1287.72	0.016724	16.83
TTL	72.91	0.016724	-105.83
ARMT1	51.90	0.016724	-95.47
MKLN1	874.71	0.016724	78.80
DENND2A	646.13	0.016724	56.33
CD151	43.82	0.016724	-86.86
RNF4	59.99	0.016724	-102.15
LRRFIP2	52.63	0.016724	-84.65
CDS2	2078.21	0.016724	33.15
EMC4	54.50	0.016724	-97.49
MDH1	40.05	0.016724	-75.94
SLC43A2	473.77	0.016724	22.95
MAP2K1	49.42	0.016724	-89.42

KXD1	43.32	0.016724	-85.97
PCNA	48.03	0.016724	-90.84
PPP4R3B	45.33	0.016724	-87.70
VPS25	37.47	0.016724	-75.20
THAP5	1414.65	0.016724	89.94
MAT2A	68.80	0.016724	-104.90
STOML2	123.98	0.016724	-83.45
COX8A	130.03	0.016724	-44.75
CCDC84	34.24	0.017244	-69.18
RPP21	36.41	0.017244	-67.82
THOC7	39.90	0.017244	-81.20
ACADVL	52.45	0.017333	-94.46
MRPL4	61.24	0.017333	-100.32
CYB5B	61.85	0.017333	-98.56
TIMM23	98.94	0.017333	-68.20
MNAT1	48.57	0.017333	-79.91
CAMLG	37.83	0.017333	-67.64
KANSL2	31.16	0.017333	-62.39
NOP10	74.80	0.017333	-111.97
TMEM18	66.90	0.017333	-76.12
GOLPH3	66.47	0.017333	-100.74
VPS29	46.22	0.017333	-85.35
RAB18	37.81	0.017333	-77.45
NUP93	52.37	0.017333	-92.90
ILVBL	42.75	0.017333	-70.04
WDR75	27.61	0.017333	-62.22
DNPH1	29.10	0.017333	-66.00
LAMTOR2	41.87	0.017333	-80.59
DDX19B	30.64	0.017333	-68.23
IFRD2	47.76	0.017333	-84.75
HEBP2	104.81	0.017333	-76.65
LOC107133515	386.34	0.017333	70.19
ZSCAN22	3458.91	0.017333	135.37
CEBPA	39.45	0.017333	-77.87
SSX2IP	33.78	0.017333	-70.90
SHKBP1	42.29	0.017333	-81.67
TOLLIP	27.34	0.017333	-59.83
OGFR	29.01	0.017333	-65.58
DPPA2	27.92	0.017333	-55.12
MRPL48	44.48	0.017333	-81.36
SUV39H2	29.32	0.017333	-55.77
CLMN	261.19	0.017333	42.36
KCTD1	24.77	0.017333	-58.07
ANKRD28	338.40	0.017333	58.53
ZKSCAN4	70.09	0.017333	-106.68
SENP3	35.55	0.017333	-73.70
MRPS6	43.98	0.017333	-82.27
TRABD	35.98	0.017333	-73.10
MRPL22	129.82	0.017333	-56.80
C4orf3	54.72	0.017333	-68.66
UBE2Q1	38.19	0.017333	-76.25
INTS12	27.86	0.017333	-63.70
RHOBTB1	54.08	0.017333	-89.62
KDM4A-AS1	882.83	0.017333	85.57
MAGI1	2803.51	0.017333	25.99

JAM3	43.24	0.017333	-75.81
AKR1A1	37.63	0.017333	-73.57
CHURC1	33.28	0.017333	-66.02
ABHD17C	35.27	0.017333	-71.70
TUBB2A	37.87	0.017333	-74.31
MZT2B	47.43	0.017333	-85.78
GLS	51.78	0.017333	-84.56
CYC1	79.78	0.017333	-56.83
RPUSD1	75.97	0.017333	-94.23
MTCH1	25.71	0.017333	-60.17
MLXIP	847.39	0.017333	39.83
SMURF1	30.40	0.017333	-66.72
CHCHD10	123.38	0.017333	-39.89
LGALS8	3783.64	0.017335	70.86
IMPA2	58.32	0.017397	-95.49
UBE2B	27.86	0.017477	-63.01
C9orf16	22.78	0.017733	-52.10
PUSL1	24.64	0.017733	-51.10
NDUFAF4	61.98	0.017890	-87.55
HMGN4	34.45	0.017890	-49.11
SH3GL1	101.97	0.018055	-47.56
PSMC6	60.63	0.018055	-90.66
TK1	42.84	0.018098	-78.04
PLEKHH3	22.05	0.018099	-54.23
DYNLT1	26.55	0.018116	-60.75
ARF4	75.12	0.018116	-93.75
YPEL3	23.87	0.018128	-54.80
ARFGAP3	24.36	0.018128	-51.44
UROD	23.79	0.018517	-56.76
CAPN1	22.67	0.018517	-54.82
DUS3L	35.38	0.018517	-65.67
ETFA	117.18	0.018517	-50.66
SH3GLB2	57.66	0.018517	-68.84
ZNF738	1467.81	0.018517	24.67
C1D	32.67	0.018890	-68.13
TNFRSF12A	30.70	0.019122	-64.69
REEP3	34.37	0.019122	-66.89
ERCC6L	29.46	0.019122	-56.18
MIR600HG	20.47	0.019143	-47.00
DDX59	108.22	0.019143	54.98
ITGB1BP1	38.00	0.019143	-46.31
VPS37C	20.15	0.019482	-49.23
EXOSC8	43.79	0.019506	-72.89
TXNDC17	512.89	0.019506	-65.55
RTKN	20.23	0.019506	-49.51
DNAJC5	29.92	0.019506	-64.02
CAPNS1	31.11	0.019506	-64.01
MMP24-AS1	37.88	0.019506	-45.15
RBM4	1011.34	0.019506	34.72
IRF2BP2	26.38	0.019506	-58.76
ALG5	53.94	0.019506	-54.19
ATP11B	36.32	0.019506	-67.32
RAB20	18.46	0.019506	-46.90
H1FX	73.56	0.019506	-60.96
GDPD3	19.00	0.019661	-45.38

HMBS	36.81	0.019661	-50.79
ATP5G1	44.94	0.019661	-71.66
ECI2	26.17	0.019661	-56.70
SLC35A4	25.12	0.019661	-55.71
RPLP0P2	493.92	0.019661	42.03
ANAPC13	16.89	0.019754	-43.29
UNC13B	34.54	0.019830	-68.82
PTCD3	145.00	0.019830	-49.20
BCAR1	54.66	0.019830	-75.15
TMCC1	311.68	0.019830	85.86
TFG	57.50	0.019830	-41.24
BLOC1S2	18.90	0.019830	-46.11
SUMO3	101.33	0.019830	-62.30
RANBP17	492.65	0.019830	100.18
GNAI2	20.92	0.019830	-50.70
SF3B5	95.10	0.019830	-51.81
EARS2	39.14	0.019830	-68.69
DVL1	57.63	0.019871	-52.53
DPP9	17.29	0.019900	-45.07
SUN3	19.70	0.020108	-49.23
PLEKHA3	23.30	0.020108	-53.56
CLDN19	229.53	0.020189	135.07
COPS9	57.51	0.020189	-42.46
TMEM164	40.59	0.020299	-70.80
TMEM258	117.09	0.020299	-52.64
NAGK	22.78	0.020299	-51.78
WDR18	28.29	0.020299	-59.47
MPP1	22.55	0.020299	-51.76
KIAA0232	1397.65	0.020299	42.42
IL34	19.83	0.020299	-49.24
RAB35	30.39	0.020299	-36.19
RPAIN	46.28	0.020419	-37.29
PAPD7	22.73	0.020617	-36.52
BTBD1	37.77	0.020708	-59.08
SDHC	23.90	0.020708	-54.64
TMEM2	828.66	0.020708	13.94
POLR2I	42.54	0.020818	-64.81
MRPS17	63.86	0.020925	-53.41
LCOR	26.22	0.021120	-53.60
RPF1	17.34	0.021344	-44.95
TBCD	25.48	0.021364	-40.00
GTF3C4	35.25	0.021507	-64.65
SBDSP1	20.58	0.021740	-49.33
GTF2F2	41.68	0.021810	-68.02
SNAPC4	26.31	0.021849	-54.40
CRAMP1	25.70	0.021849	-39.19
RHOG	36.72	0.021849	-44.11
EBAG9	27.60	0.021924	-55.06
OTUD6B-AS1	24.39	0.021940	-53.30
ARMC1	48.65	0.021951	-43.31
PPP1R15A	29.22	0.022116	-34.52
POLR2C	15.38	0.022127	-41.03
ALG2	36.16	0.022276	-65.73
G3BP2	147.40	0.022691	-58.21
SLC52A2	35.41	0.022691	-57.91

DIMT1	933.05	0.022691	41.44
SETD9	219.43	0.022818	59.54
CGGBP1	27.89	0.022948	-57.51
FLII	37.91	0.022948	-30.46
GCSH	17.10	0.022989	-43.73
UBE2R2	39.59	0.023182	-40.35
PPP6C	18.97	0.023182	-45.58
ZNF518A	155.80	0.023196	-31.80
ZNF276	664.68	0.023427	16.05
POLH	703.28	0.023450	26.78
TCF7L1	19.87	0.024370	-44.99
MCRIP2	85.81	0.024370	-38.15
CRCP	51.00	0.024370	-45.79
SELENOH	123.22	0.024720	-49.32
GLOD4	65.22	0.024735	-28.67
SCIMP	1883.73	0.024789	64.80
TTC37	34.82	0.025016	-46.81
MORC3	68.28	0.025224	-47.57
PIP4K2C	13.39	0.025564	-36.95
FRG1	47.91	0.025965	-30.18
LACTB2	38.78	0.026092	-40.44
ITPA	35.96	0.026143	-56.69
APBB1	31.57	0.026219	-43.84
DNAJC24	14.33	0.026527	-38.75
BOLA3	12.88	0.026873	-35.95
GOT1	34.92	0.026873	-28.87
NPM3	20.71	0.026941	-33.20
RYK	13.09	0.026941	-36.35
HPDL	23.41	0.026941	-49.37
JOSD1	17.96	0.027081	-41.70
BIRC2	28.80	0.027339	-25.23
UBIAD1	1393.95	0.027729	22.55
NBEAL2	12.81	0.027919	-35.73
HIBADH	28.55	0.028123	-48.74
USF2	42.92	0.028123	-26.30
DIABLO	98.77	0.028534	-25.84
ARL15	367.88	0.028570	47.05
PPP1CC	211.24	0.028606	-18.87
DARS	104.48	0.028606	-30.24
SMYD5	14.88	0.029042	-38.28
THOC6	14.32	0.029053	-37.82
CMTM4	34.80	0.029159	-28.29
JPT1	900.44	0.029159	-11.83
TSPAN6	12.67	0.029213	-35.17
JMJD8	92.74	0.029598	-48.50
UQCC1	19.62	0.029598	-41.95
COMMD9	33.10	0.029629	-28.10
ANAPC7	493.22	0.029629	13.02
EIF4H	240.19	0.029671	-42.85
CENPT	96.35	0.030196	-45.00
PCYT1B	811.62	0.030211	70.78
FZD3	534.74	0.030219	16.61
GRTP1	52.71	0.030758	-35.63
PCYT2	63.60	0.030758	-45.58
LYPLA1	14.36	0.030758	-36.38

HPS5	660.23	0.030758	58.55
KDELR2	110.02	0.030802	-48.65
POLB	33.06	0.030824	-25.41
POTEM	424.86	0.031071	30.25
GLMN	21.85	0.031071	-19.10
PRDX2	66.13	0.031071	-27.17
SNAPC5	238.95	0.031071	39.64
TDGF1P3	2239.46	0.031071	63.38
NEK6	10.91	0.031071	-30.65
ERBB2	12.85	0.031071	-34.81
PSMD6	76.69	0.031522	-30.74
CTDSP1	47.23	0.031736	-31.34
COPE	29.18	0.031736	-35.35
FRAT2	58.98	0.032082	-41.08
RASSF2	2414.97	0.032082	52.95
SOCS7	31.08	0.032089	-25.81
PUS3	15.75	0.032294	-36.44
VPS26B	19.78	0.032294	-29.24
AKTIP	10.73	0.032306	-30.01
DDX39A	90.62	0.032306	-34.27
FBRSL1	43.77	0.032423	-37.66
ATXN2L	12.43	0.032648	-33.58
RMND1	1080.52	0.033141	38.49
ABHD11	11.94	0.033141	-32.93
LIAS	31.63	0.033141	-25.26
SLC25A33	29.20	0.033141	-41.71
NDUFB3	62.89	0.033282	-29.76
SNX3	55.13	0.033282	-33.00
SFT2D1	46.46	0.033369	-35.58
ACTL6A	39.42	0.033369	-25.32
GORASP2	157.45	0.033516	-47.43
HIF3A	507.13	0.033516	59.96
MRPL44	30.31	0.033516	-23.48
COMTD1	66.06	0.033516	-44.09
DDIAS	14.71	0.033745	-24.64
ZNF721	22.53	0.034056	-26.89
ZNF92	53.24	0.034099	-34.62
TRUB2	12.81	0.034449	-33.33
RBM14	83.90	0.034449	-31.94
MPDU1	13.64	0.034570	-33.68
STEAP3	152.64	0.034711	28.87
MTRNR2L10	1102.43	0.034779	6.51
EIF2B1	22.47	0.034804	-39.16
GTSF1	57.77	0.034804	-30.64
CTU2	11.98	0.034804	-32.33
NCOR2	46.25	0.034945	-28.50
RNF111	36.05	0.035263	-29.42
SMIM26	27.45	0.035568	-38.79
RAB40C	15.00	0.035568	-34.23
ZNF557	262.69	0.035704	45.98
SPARC	347.13	0.035704	-28.96
CCDC168	4341.02	0.035914	28.80
ERCC3	10.35	0.035914	-29.73
TERF2	19.46	0.035914	-35.79
TRIM28	192.85	0.035914	-24.77

LOC646214	453.14	0.035914	21.32
METTL1	27.24	0.036180	-21.93
GLT8D1	12.96	0.036541	-32.98
AP1AR	21.89	0.036541	-19.44
RAB5C	13.38	0.037063	-32.58
ROCK1P1	1013.14	0.037227	13.26
MAP2K2	35.58	0.037227	-20.10
PSMD3	34.92	0.037263	-20.80
EPC2	43.88	0.037934	-31.41
C19orf53	87.87	0.038060	-23.70
ZNRF2	232.54	0.038181	58.36
ZGRF1	693.01	0.038352	28.25
PITPNA	35.12	0.038352	-29.41
IPO4	24.52	0.038768	-28.60
TMEM256	13.20	0.038768	-31.79
CTU1	18.99	0.039978	-23.54
CTPS1	35.00	0.040014	-23.52
SMIM12	46.53	0.040014	-22.06
E4F1	41.40	0.040218	-21.20
C9orf64	15.68	0.040396	-14.78
XAGE3	11.25	0.040396	-30.60
C5orf42	387.91	0.040505	29.43
PKHD1	341.86	0.040592	34.53
LRRC8E	591.30	0.040816	42.51
DYNLRB1	28.14	0.040816	-20.88
EXOSC6	91.94	0.041146	-40.26
CYB5R3	49.67	0.041928	-23.64
RASSF1	26.47	0.042211	-22.75
GLUL	112.44	0.042211	-34.99
BLOC1S3	28.89	0.042376	-18.17
METTL6	322.80	0.043107	16.13
AKIRIN2	16.59	0.043189	-31.34
HK2	91.90	0.043189	-28.17
DECR2	51.80	0.043189	-22.71
TRIM11	89.44	0.043371	-38.26
NUDT5	78.19	0.043697	-38.53
OS9	614.04	0.043697	18.13
BUD31	69.83	0.043759	-21.81
FBXO34	423.49	0.043915	23.87
EXOSC3	220.86	0.043915	-44.15
CREB1	109.98	0.044597	-38.92
GNG10	8.62	0.044597	-25.77
PRELID3B	25.05	0.044713	-31.68
SORBS1	35.28	0.045289	-22.18
NARS2	17.16	0.045289	-15.71
FBXO45	89.23	0.045356	-32.14
MCL1	82.94	0.045658	-24.43
XAB2	17.01	0.045658	-15.80
SH2D4A	119.34	0.045658	-33.30
KIAA1143	47.69	0.045658	-23.72
UBE2H	49.64	0.045658	-24.16
GRWD1	164.10	0.046128	-40.81
ZNF286A	849.46	0.046212	16.42
TMEM165	52.93	0.046283	-33.98
ZCCHC10	32.18	0.046283	-22.12

NUS1	46.24	0.046283	-21.64
ZNF33A	43.50	0.046358	-28.42
OAT	30.00	0.046358	-29.16
ATXN3	1427.44	0.046358	30.55
ANAPC10	15.06	0.046358	-29.15
MIR22HG	54.84	0.046399	-33.94
LPAR2	36.11	0.046485	-28.30
ZNF429	1013.55	0.046485	24.93
RRP9	126.37	0.046751	-41.25
KIF3B	634.17	0.046751	35.53
BCKDK	39.98	0.047372	-24.92
FOXH1	30.11	0.047372	-19.56
CISD1	214.37	0.047385	-37.69
POLR1E	37.33	0.047385	-17.68
RAB25	79.88	0.047385	-29.47
CAMSAP3	27.99	0.047385	-18.78
H2AFX	52.78	0.047555	-24.06
C20orf27	16.32	0.047915	-28.12
ABHD5	609.26	0.047915	16.81
AP5Z1	29.58	0.047938	-19.84
SUMO1	113.30	0.048031	-33.65
SYPL1	28.25	0.048031	-14.89
COX7B	331.98	0.048341	-14.75
COX16	113.54	0.048452	-20.37
GRB10	98.38	0.048714	-35.98
SLC25A1	261.84	0.048714	-23.37
BRK1	20.97	0.049835	-21.80

TABLE S5: DIFFERENTIAL EXPRESSION OF GENES – TRISOMY 16 VS MONOSOMY 16 SAMPLES

Gene ID	Total counts	FDR step up	Fold change
KLK7	3.26	0.00000	157.67
XAGE3	8.22	0.00000	200.94
GOLGA2P7	2.17	0.00000	292.83
PHF12	29.66	0.00000	5.89
RRS1-AS1	91.06	0.00001	154.39
NPIPB13	4.31	0.00001	10.28
C8orf82	24.14	0.00001	11.39
PAQR6	4.38	0.00001	8.06
CDR2	10.95	0.00001	13.50
RHOBTB1	64.48	0.00005	33.37
LOC643802	25.51	0.00005	934.66
CENPT	116.99	0.00005	26.83
GINS2	90.56	0.00010	12.50
HCFC1R1	10.31	0.00010	15.16
LOC100652768	1.00	0.00010	12.91
FAM220A	14.54	0.00012	16.98
DSG4	53.10	0.00018	-8.06
FAM3A	22.82	0.00020	674.47
FGFR2	335.75	0.00024	-286.74
GDF15	1091.87	0.00024	-411.31
MT1L	1.73	0.00026	211.77
VAMP4	2.00	0.00026	182.49
KLK2	5.96	0.00026	257.99
CD79B	0.95	0.00033	3108.25
ZNF571	0.57	0.00033	1874.41
ZBTB20-AS3	0.19	0.00033	628.52
CALY	0.78	0.00033	414.09
GNRH1	0.69	0.00035	261.85
TIGD3	0.40	0.00036	214.83
PLAU	14.59	0.00039	6590.92
PIGQ	5.49	0.00039	58.32
ZNF19	2.50	0.00039	9292.23
COG4	19.07	0.00040	9.19
SERPING1	1.06	0.00041	2647.86
ASF1B	4.77	0.00045	16.88
ABHD17C	108.88	0.00045	7.38
CXorf40B	11.50	0.00046	4.64
RHOXF1P1	1.34	0.00046	152.95
PLEKHF1	26.68	0.00047	10.87
RRAGA	11.01	0.00052	29.77
DDX19B	54.55	0.00054	8.35
LMF1	12.60	0.00057	631.99
SLC25A46	4.06	0.00059	10.32
EARS2	59.63	0.00063	8.77
CALML4	81.79	0.00066	7.59

CDK10	75.49	0.00069	8.52
HIST4H4	20.07	0.00073	555.54
TMEM158	5.71	0.00074	19.13
MCTP2	7.86	0.00076	51.45
LOC101929516	5.96	0.00088	55.30
ZNF252P-AS1	55.80	0.00088	527.85
TRAF3IP2	33.81	0.00092	24.43
LINC00167	59.73	0.00092	539.89
SNX11	42.31	0.00099	12.80
BCKDK	54.47	0.00115	14.36
GREM2	3.75	0.00122	29.51
ADCY8	2.05	0.00122	16.55
FIGN	156.82	0.00124	6.13
VPS9D1-AS1	21.09	0.00125	63.15
LINC01087	13.19	0.00135	98.08
PWWP2B	58.22	0.00137	5.56
RPP25L	12.09	0.00146	56.42
SRD5A3	36.15	0.00150	10.40
TUBB2B	8.22	0.00155	17.15
N4BP1	45.94	0.00157	14.16
ZNF528	9.07	0.00157	-1.20
SLC52A1	6.74	0.00165	1.92
THOC6	21.69	0.00165	9.05
EYA4	4.52	0.00167	-10.21
GEMIN6	52.40	0.00168	5.09
CBWD3	0.63	0.00176	97.01
DUSP11	29.84	0.00182	-3.97
FOXC1	0.69	0.00189	23.96
CFAP57	25.09	0.00195	-13.05
ARFGAP3	38.34	0.00197	14.07
PUS7L	31.97	0.00202	5.75
ZNF782	41.20	0.00207	4.44
D21S2088E	4.88	0.00215	26.85
CTSH	23.51	0.00228	62.16
GRIN2D	4.00	0.00230	2305.13
MYC	50.36	0.00233	21.08
RAB20	42.46	0.00240	3.97
ZNF33A	131.92	0.00255	4.89
PEX11B	36.53	0.00262	-26.83
MTAP	98.08	0.00266	5.49
PAXIP1	63.81	0.00278	14.75
ARHGAP17	47.74	0.00283	9.83
SH3PXD2B	62.03	0.00285	16.36
CDIPT	100.37	0.00290	10.72
TMPRSS2	61.40	0.00291	312.47
ETAA1	23.60	0.00292	30.80
DYRK1B	5.70	0.00294	4.17

CREBBP	297.91	0.00305	5.93
DNM1P41	2.33	0.00305	63.09
CLCN7	69.24	0.00311	5.72
SMCR5	23.92	0.00317	-30.92
C21orf91	11.23	0.00317	50.36
LILRA5	41.86	0.00318	-10.63
GATA6-AS1	10.56	0.00321	2.85
MAGEB2	4.67	0.00325	83.82
MVB12A	20.19	0.00326	36.89
ATP6V1G2-DDX39B	0.02	0.00330	4.47
SLC43A3	16.32	0.00334	3884.07
GPR137B	3.56	0.00351	47.28
RMI2	17.46	0.00354	1.97
GDF15	255.69	0.00381	-5.67
LOC101928105	0.69	0.00402	195.83
XRCC1	24.70	0.00409	8.71
HSPBAP1	11.84	0.00422	-18.40
UBR7	49.62	0.00430	9.50
VASH1-AS1	31.48	0.00456	7.41
METTL27	4.27	0.00459	5.16
ARL8A	14.91	0.00461	10.20
MAP9	67.51	0.00461	3.91
EML2	3.58	0.00462	3.72
UCHL1	37.52	0.00465	3.33
COTL1	43.25	0.00478	81.69
CPT1A	50.83	0.00494	-6.73
PAQR4	77.79	0.00505	295.68
SATB1	12.13	0.00515	2.89
SSX2B	0.29	0.00527	15.52
CA11	2.05	0.00540	115.79
SH2B2	37.40	0.00541	25.22
ATAT1	14.21	0.00543	6.42
ASIC3	6.75	0.00547	295.82
DENND5B-AS1	2.39	0.00549	10.68
LOC105375650	16.29	0.00554	41.57
USP31	436.45	0.00561	88.46
N4BP1	102.60	0.02025	141.18
MGAT4EP	5683.84	0.02827	-175.19
PLEKHA5	793.84	0.04982	-19.17
RHOBTB1	122.66	0.04982	147.49
DDX19B	144.10	0.04982	122.03
